# SIRT5‐Mediated Desuccinylation of ACAA2 Protects Against Calcium Oxalate‐Induced Kidney Injury by Regulating Fatty Acid Oxidation to Suppress Mitochondrial Oxidative Injury and Ferroptosis

**DOI:** 10.1002/advs.77703

**Published:** 2026-09-27

**Authors:** Qinhong Jiang, Caitao Dong, Yuchen Liu, Yuanquan Lou, Wenlong Lin, Sheng Li, Ziqi He

**Affiliations:** ^1^ Department of Urology Renmin Hospital of Wuhan University Wuhan University Wuhan China; ^2^ Hubei Provincial Clinical Research Center for Urolithiasis Renmin Hospital of Wuhan University Wuhan China; ^3^ Shenzhen Institute of Translational Medicine Health Science Center The First Affiliated Hospital of Shenzhen University Shenzhen University Shenzhen China; ^4^ Department of Urology Zhongnan Hospital of Wuhan University Wuhan University Wuhan China; ^5^ Department of Urology Cancer Precision Diagnosis and Treatment and Translational Medicine Hubei Engineering Research Center Zhongnan Hospital of Wuhan University Wuhan China

**Keywords:** beta oxidation, calcium oxalate kidney stones, fatty acid, fatty acid beta‐oxidation, kidney stone disease, lipidomics, lipotoxicity, polyunsaturated fatty acid, SIRT5, sirtuin

## Abstract

Renal tubular epithelial cells (RTECs) are highly metabolically active and vulnerable to pathological stress. Calcium oxalate (CaOx) crystals disrupt lipid metabolism and damage RTECs, thereby promoting nephrolithiasis. We found that SIRT5, a highly expressed desuccinylase in RTECs, was markedly reduced in kidneys from patients with CaOx stones and in mouse models. Multi‐omics analyses integrating succinylomics, lipidomics, and proteomics showed that SIRT5 maintains lipid metabolic homeostasis by desuccinylating key fatty acid oxidation (FAO) enzymes. SIRT5 deficiency impaired FAO, causing polyunsaturated fatty acid (PUFA) accumulation, phospholipid remodeling, and ferroptosis. Oxidative membrane damage subsequently increased crystal adhesion, establishing a vicious cycle of crystal deposition and tubular injury. Acetyl‐CoA acyltransferase 2 (ACAA2) was identified as a functional SIRT5 substrate. The K13R mutation reduced ACAA2 succinylation and preserved its activity, whereas K13E impaired the protective effect of SIRT5 overexpression against CaOx‐induced injury. Collectively, our findings define a SIRT5‐ACAA2 axis linking impaired FAO to lipid peroxidation, ferroptosis, and crystal adhesion, highlighting this pathway as a potential therapeutic target for CaOx nephrolithiasis.

## Graphical abstract

The SIRT5/ACAA2 axis maintains lipid metabolic homeostasis in RTECs by promoting ACAA2 desuccinylation and preserving its stability and enzymatic activity, thereby sustaining FAO and limiting PUFA and PUFA‐containing phospholipid accumulation. Under CaOx stress, SIRT5 downregulation impairs FAO, promotes oxidative stress and ferroptosis, and enhances crystal adhesion. Pharmacological activation of SIRT5 may therefore provide a potential therapeutic strategy for CaOx nephrolithiasis.

## 1 Introduction

Kidney stone is a highly prevalent and recurrent urological disease, with a postoperative recurrence rate reaching up to 50% within five years [1, 2]. The recurrent and progressive nature of the disease often leads to irreversible renal fibrosis and functional decline, conditions that cannot be adequately addressed by lithotripsy or surgical extraction alone [3]. Among all stone types, calcium oxalate (CaOx) stones account for approximately 75% of cases and constitute the primary component of most urinary calculi [4]. The formation of CaOx stones is a dynamic and multi‐step crystallization process initiated by crystal nucleation, in which inflammation, cellular injury, and metabolic disturbances serve as key driving forces [5, 6]. Renal tubular epithelial cells (RTECs), as essential structural components of the kidney, are metabolically active and highly vulnerable to stress. Metabolic dysfunction and cellular injury of RTECs induced by CaOx crystals are considered critical initiating events in stone formation [6]. Thus, elucidating the mechanisms underlying RTEC injury and identifying potential therapeutic targets in CaOx nephrolithiasis are of considerable clinical significance.

Metabolic reprogramming, particularly lipid metabolic remodeling, has been increasingly recognized as a central contributor to the pathogenesis and progression of kidney stones [7, 8]. Under physiological conditions, robust fatty acid oxidation (FAO) provides essential energy support for cells with high metabolic demands, such as RTECs. However, emerging evidence indicates that under CaOx crystal exposure, FAO becomes impaired in RTECs, which in turn exacerbates stone formation and renal injury, thereby revealing a potential therapeutic avenue [9]. Furthermore, defective FAO results in intracellular accumulation of unoxidized polyunsaturated fatty acids (PUFAs). Once incorporated into phospholipids, they become susceptible substrates for free radical‐induced oxidation, triggering ferroptosis, a form of non‐apoptotic cell death driven by iron‐catalyzed peroxidation of membrane phospholipids [10]. Accumulating evidence suggests that ferroptosis of RTECs under CaOx exposure plays a crucial role in stone formation, and its inhibition significantly reduces crystal deposition in rat models of nephrolithiasis [11]. Therefore, FAO dysfunction induced by CaOx crystal exposure may further promote lipid remodeling and ferroptosis in RTECs, jointly contributing to the development of CaOx kidney stones.

Succinylation is a recently identified post‐translational modification that targets lysine residues and is widely distributed across various mitochondrial metabolic enzymes [12, 13]. Previous studies have reported significantly elevated succinate levels in multiple kidney diseases, which are closely associated with metabolic disturbances and reactive oxygen species (ROS) generation [14, 15, 16]. Sirtuin 5 (SIRT5), an NAD^+^‐dependent desuccinylase, serves as a key regulator of protein succinylation and exerts important epigenetic control over diverse metabolic processes [13]. Recent evidence has indicated that SIRT5 regulates lipid metabolism by modulating the activity of FAO‐related enzymes [17, 18], indicating that it may function as a critical node linking lipid metabolic reprogramming to pathogenesis. Nevertheless, the role and underlying mechanisms of SIRT5 in CaOx nephrolithiasis remain largely unexplored.

In this study, we demonstrate that SIRT5 abundance is reduced in RTECs following CaOx exposure. SIRT5 deficiency exacerbates mitochondrial oxidative stress and ferroptosis, and further aggravates crystal deposition and renal functional impairment. Mechanistically, SIRT5 enhances the enzymatic activity of Acetyl CoA Acyltransferase 2 (ACAA2), a key FAO enzyme, by mediating desuccinylation at the K13 residue. This modification reduces PUFA accumulation and mitigates oxidative damage to plasma membrane phospholipids in RTECs, thereby alleviating CaOx crystal adhesion and exerting a protective effect. Collectively, our findings elucidate a novel regulatory mechanism and highlight the therapeutic potential of targeting SIRT5 in CaOx kidney stone disease.

## 2 Materials and Methods

### 2.1 Animal Experiments

#### 2.1.1 Mouse

All experimental procedures were approved by the Animal Ethics Committee of Renmin Hospital of Wuhan University (Approval No. 202400280F). Wild‐type (WT) mice on a C57BL/6J background were purchased from the Hubei Provincial Center for Disease Control and Prevention. *Sirt5* ^fl/fl^ mice and *Cdh16*‐cre mice were obtained from Cyagen Biosciences (Guangzhou, China), and *Cdh16*‐cre *Sirt* ^fl/fl^ mice were generated through crossbreeding. Genotyping was performed by PCR analysis of DNA extracted from mouse tail tissue. All animals were housed in a controlled environment maintained at 20°C–22°C with a 12 h light/dark cycle and had free access to food and water.

#### 2.1.2 Administration

The animal experiments were conducted in three independent parts. In each part, five mice were included in each experimental group (n = 5 per group). The experimental groups and treatment regimens were as follows:

Part I: *Saline group*: mice received a daily intraperitoneal (i.p.) injection of 100 µL saline for 14 consecutive days. *Glyoxylate (Gly) group*: mice received a daily i.p. injection of Gly (80 mg/kg; MedChemExpress, Wuhan, China) for 14 consecutive days.
Part II: *Sirt5^fl/fl^ + Saline group*: Sirt5^fl/fl^ mice received a daily i.p. injection of 100 µL saline for 14 consecutive days. *Sirt5^−/−^ + Saline group*: Sirt5^−/−^ mice received a daily i.p. injection of 100 µL saline for 14 consecutive days. *Sirt5^fl/fl^ + Gly group*: Sirt5^fl/fl^ mice received a daily i.p. injection of Gly (80 mg/kg) for 14 consecutive days. *Sirt5^−/−^ + Gly group*: Sirt5^−/−^ mice received a daily i.p. injection of Gly (80 mg/kg) for 14 consecutive days.
Part III: Control group: mice received a daily i.p. injection of 100 µL saline with DMSO and SBE‐β‐CD for 14 consecutive days. MC3138 control group: mice received a daily i.p. injection of 100 µL MC3138 dissolved in 10% DMSO and 90% SBE‐β‐CD saline solution (10 mg/kg; MedChemExpress) for 14 consecutive days. Gly group: mice received a daily i.p. injection of Gly (80 mg/kg) for 14 consecutive days. Gly + MC3138 group: mice received daily i.p. injections of Gly (80 mg/kg) and MC3138 (10 mg/kg) for 14 consecutive days.

After the intervention, blood was collected from the retro‐orbital venous plexus, and serum was separated by centrifugation. The mice were then euthanized, and kidney tissues were harvested for subsequent analysis.

### 2.2 Cell Experiments

#### 2.2.1 Cell Culture

Human renal tubular epithelial cells (HK‐2) were obtained from Procell Life Science & Technology Co., Ltd. (Wuhan, China). The cells were cultured in Dulbecco's Modified Eagle Medium (DMEM) /Nutrient Mixture F‐12 (Procell Life Science & Technology Co., Ltd.) supplemented with 10% fetal bovine serum (FBS) and 1% antibiotics (penicillin and streptomycin) at 37°C in a humidified atmosphere containing 5% CO_2_.

#### 2.2.2 Administration

To replicate the crystal‐induced cellular injury observed in calcium oxalate (CaOx) kidney stones, cells were allowed to reach 70%–80% confluence and then treated with 1.5 mM calcium oxalate (CaOx) solution (Solarbio, Beijing, China) for 24 h, establishing an in vitro model of crystal‐induced cellular injury.

#### 2.2.3 Cell Transfection

Lentiviral (LV) vectors for SIRT5 gene knockdown and overexpression, along with the corresponding negative controls, were prepared by Shanghai Jino Biolab Co., Inc. (Shanghai, China). When HK‐2 cells reached 50% confluence, the lentiviral vectors containing either oe‐SIRT5 or sh‐SIRT5 were transfected using a transfection reagent. Stable transfected cell lines were then selected using medium supplemented with 2 µg/ml puromycin. Wild‐type and point mutation ACAA2 plasmids (K13R, K137R, K270R, K13E, K270E) were constructed by PTM BioLab Co., Ltd. (Hangzhou, China) and transfected into HK‐2 cells using Lipofectamine 2000 (Thermo Fisher Scientific Inc., MA, USA) according to the manufacturer's instructions.

### 2.3 Targeted Lipidomic Profiling

After collection, cell samples were stored at −80°C. The samples were thawed on ice, and all subsequent procedures were performed on ice. A lipid extraction solution containing an internal standard (methyl tert‐butyl ether:methanol = 3:1, v/v) was added to the samples at a volume of 1 mL. After vortex mixing, 200 µL of water was added, and the samples were centrifuged at 12 000 r/min for 10 min at 4°C. The supernatant was collected and concentrated to dryness, then re‐dissolved in 200 µL of lipid resuspension solution (acetonitrile:isopropanol = 1:1, v/v). The mixture was centrifuged at 12 000 r/min for 3 min, and the supernatant was used for liquid chromatography‐tandem mass spectrometry (LC‐MS/MS) analysis. Chromatographic and mass spectrometric data were collected using UPLC (ExionLC AD, PerkinElmer, MA, USA) coupled with a tandem mass spectrometer (QTRAP 6500+, PerkinElmer). Following data acquisition, qualitative analysis was performed based on the Metware database. Mass spectrometric data processing was carried out using Analyst 1.6.3 software.

### 2.4 4D‐Label Free Quantitation (4D‐LFQ) Proteomic Analysis

After the corresponding interventions, HK‐2 cells were collected by removing the culture medium and storing the cell samples at −80°C. Prior to analysis, all samples underwent enzymatic digestion. Protein abundance was evaluated using LC‐MS, and the resulting MS/MS data were processed with the MaxQuant search engine (v.1.6.15.0). Proteins identified and quantified in this study were further analyzed based on their functions and characteristics. Differentially expressed proteins (DEPs) were functionally annotated and enriched using Kyoto Encyclopedia of Genes and Genomes (KEGG) pathway analysis and Gene Ontology (GO) annotations. Gene set enrichment analysis (GSEA) was performed to identify significantly altered gene sets.

### 2.5 Single Cell Analysis

The single‐cell RNA sequencing data used for analysis were obtained from the GSE231569 dataset in the Gene Expression Omnibus (GEO) database (http://www.ncbi.nlm.nih.gov/geo/), which contains single‐nucleus profiling data of kidney tissues from healthy individuals and patients with kidney stones. Following quality control, normalization, and batch effect correction, clustering analysis and visualization were performed using the Uniform Manifold Approximation and Projection (UMAP) method. Major cell populations were annotated using the SingleR package, and epithelial cell subpopulations were further annotated based on the Cell Marker database (http://bio‐bigdata.hrbmu.edu.cn/CellMarker/).

### 2.6 LC‐MS/MS Analysis‐ of Peptide‐ Succinylation

After HK‐2 cell intervention, the culture medium was removed, and the cells were collected. LC‐MS/MS analysis of peptide succinylation was performed by PTM BioLab Co., Ltd. (Hangzhou, China). Protein concentration was determined using a BCA kit. Following trypsin digestion, the peptides were desalted using C18 solid‐phase extraction columns. The tryptic digests were incubated with anti‐succinyllysine antibody‐conjugated beads (PTM BioLab Co., Ltd.) for enrichment of post‐translational modifications, and the resulting peptides were desalted with C18 ZipTips (Millipore). Subsequently, the peptides were subjected to LC‐MS/MS analysis, and the mass spectrometry data were processed using the MaxQuant search engine (v.1.6.15.0) followed by bioinformatics analysis.

### 2.7 Western Blotting

Total protein was extracted from cells or tissues using radioimmunoprecipitation assay (RIPA) buffer (Solarbio). Protein samples were separated by 10%–12% sodium dodecyl sulfate–polyacrylamide gel electrophoresis (SDS‐PAGE) and transferred onto polyvinylidene difluoride (PVDF) membranes (Bio‐Rad, CA, USA), which were then blocked with rapid blocking buffer (Epizyme, MA, USA). After washing with Tris‐buffered saline containing Tween 20 (TBST), the membranes were incubated overnight at 4 °C with primary antibodies against sirtuin 5 (SIRT5), glyceraldehyde‐3‐Phosphate dehydrogenase (GAPDH), NAD(P)H quinone dehydrogenase 1 (NQO‐1), heme Oxygenase 1 (HMOX1), kidney injury molecule 1 (KIM‐1), neutrophil gelatinase‐associated lipocalin (NGAL), Acyl‐CoA synthetase long chain family member 4 (ACSL4), transferrin Receptor (CD71), solute carrier family 7 member 11 (xCT), ferritin heavy chain 1 (FTH1), glutathione peroxidase 4 (GPX4), Acetyl‐CoA acyltransferase 2 (ACAA2), annexin A1 (ANXA1), annexin A2 (ANXA2), carnitine palmitoyl transferase 1A (CPT1A), Hemagglutinin (HA), Flag, and succinylation (detailed antibody information is provided in Table S1). Following TBST washes, the membranes were incubated with secondary antibodies for 1 h at room temperature. After a final TBST wash, protein bands were visualized using an Odyssey infrared imaging system (LI‐COR, NE, USA). The grayscale intensity of the images was quantified using ImageJ software (version 1.53S).

### 2.8 Immunoprecipitation and Coimmunoprecipitation (Co‐IP)

Cells were lysed using IP lysis buffer (Beyotime, Shanghai, China), and lysates were centrifuged at 12 000 r/min for 15 min at 4°C to obtain the protein extracts. Lysates were incubated overnight at 4°C with 2 µg of antibody (IgG/ACAA2/succinylation). After washing the beads three times with IP lysis buffer, they were incubated with protein A/G agarose beads (Selleck, TX, USA) overnight at 4°C. Following three additional washes with IP lysis buffer, 5× loading buffer was added, and samples were boiled at 95°C for 5 min to elute proteins. The resulting proteins were analyzed by western blotting.

### 2.9 Cell Viability Assay

Cell viability was assessed using the Cell counting kit‐8 (CCK‐8) assay (Biosharp, Beijing, China). Cells were seeded into a 96‐well plate, and when the confluence reached 70%, the corresponding interventions were applied. After intervention, the culture medium was removed, and 100 µL of CCK‐8 working solution, diluted with basal medium, was added to each well. The plate was then incubated at 37°C in the dark for 2 h. Finally, absorbance at 450 nm was measured using a microplate reader (PerkinElmer).

### 2.10 Detection of Antioxidant and Oxidative Stress Markers

The levels of total antioxidant capacity (T‐AOC), lactate dehydrogenase (LDH), superoxide dismutase (SOD), malondialdehyde (MDA), and glutathione (GSH) were measured according to the manufacturer's instructions using the following kits: T‐AOC assay kit (Nanjing Jiancheng, Nanjing, China), LDH assay kit (Nanjing Jiancheng), SOD assay kit (Nanjing Jiancheng), MDA assay kit (Nanjing Jiancheng), GSH assay kit (Nanjing Jiancheng). Absorbance was measured using a microplate reader (PerkinElmer) at the following wavelengths: 450 nm for LDH and SOD, 405 nm for T‐AOC and GSH, and 530 nm for MDA.

### 2.11 Detection of Reactive Oxygen Species (ROS) Level

After cellular interventions, the culture medium was removed, and cells were washed twice with PBS. Cells were then incubated with 2,7‐dichlorodihydrofluorescein diacetate (Dojindo, Tokyo, Japan) working solution, diluted in basal medium, at 37°C in the dark for 30 min. Following incubation, cells were washed with basal medium. Intracellular ROS levels were measured using a flow cytometer (Beckman Coulter, CA, USA), and fluorescence intensity was analyzed with CytExpert software.

### 2.12 Lipid Peroxidation Detection and Subcellular Localization

Lipid peroxidation levels were assessed using BODIPY 581/591 C11 (Thermo Fisher Scientific Inc.). After cell intervention and washing, cells were incubated with 10 µmol/L probe working solution at 37°C for 30 min. Fluorescent signals reflecting lipid peroxidation were observed using a confocal microscope (Olympus, Tokyo, Japan), and quantitative analysis was performed using ImageJ software (version 1.53S). For plasma membrane staining, the Cell Plasma Membrane Staining Kit (Beyotime) was used, with DID working solution diluted in PBS, and cells were incubated at 37°C in the dark for 15 min. Mitochondria were stained using Mito‐Tracker Deep Red 633 (Beyotime), diluted 1:1000 in Hanks’ Balanced Salt Solution (HBSS), and incubated at 37°C for 10 min.

### 2.13 Mitochondrial Membrane Potential Measurement

After cell intervention and washing, JC‐1 working solution (Beyotime) diluted in staining buffer and basal medium was added to each well, and the cells were incubated at 37°C for 20 min. After incubation, the working solution was discarded, and cells were washed twice with cold 1× JC‐1 staining buffer. Fluorescence images were immediately captured using an inverted fluorescence microscope (Olympus), and fluorescence intensity was quantified using ImageJ software (version 1.53S).

### 2.14 Mitochondrial ROS Detection

Mitochondrial ROS levels were detected using the MitoSOX probe (MedChemExpress). After cell intervention and washing, cells were incubated with 5 µM MitoSOX working solution diluted in PBS at 37°C in the dark for 10 min. Cells were then washed three times with cold PBS, and fluorescence was observed using a confocal microscope (Olympus). Quantitative analysis of fluorescence intensity was performed using ImageJ software (version 1.53S).

### 2.15 Detection of Cell Apoptosis

Cell apoptosis was assessed using a V‐fluorescein isothiocyanate (FITC)/propidium iodide (PI) apoptosis detection kit (Elabscience, Wuhan, China). After treatment, cells were incubated with Annexin V‐FITC and PI according to the manufacturer's instructions. Fluorescence signals were detected using a flow cytometer (Beckman Coulter, CA, USA), and data were analyzed using CytExpert software.

### 2.16 Detection of Acetyl Coenzyme A (CoA)

Cellular acetyl‐CoA levels were measured using an Acetyl CoA enzyme‐linked immunosorbent assay (ELISA) Kit (Elabscience). After treatment, cells were washed and lysed by ultrasonication. The cell lysates were added to the ELISA plate and incubated at 37°C for 90 min. Subsequently, biotinylated antibody working solution, HRP‐conjugated reagent, substrate solution, and stop solution were added sequentially according to the manufacturer's instructions. Absorbance was measured at 450 nm using a microplate reader (PerkinElmer).

### 2.17 Detection of Adenosine Triphosphate (ATP)

Cellular ATP levels were measured using an Enhanced ATP Chemiluminescence Assay Kit (Elabscience). Cells were washed and collected according to the manufacturer's instructions, followed by the addition of the detection working solution. Luminescence was immediately measured using a microplate reader (PerkinElmer).

### 2.18 Crystal‐Cell Adhesion

After treatment, cells were washed with HBSS on a shaker at room temperature for 2 min at 150 rpm, and this step was repeated three times. Subsequently, cells were incubated with Sirius Red S working solution for 30 min at room temperature in the dark, followed by three additional washes with HBSS. The adhesion of CaOx crystals was observed under white light using an inverted microscope (Olympus), and quantitative analysis was performed using ImageJ software (version 1.53S).

### 2.19 Cell Adhesion Ability Assay

96‐plates were pre‐coated with 10 µg/mL fibronectin (ABclonal, Wuhan, China) at room temperature for 1 h. After removing the coating solution, 200 µL of heat‐denatured 1% bovine serum albumin (BSA) was added, followed by incubation at 37°C for 1 h. The BSA solution was then removed, and the plates were washed with basal medium. After treatment, cells were trypsinized and seeded into the pretreated 96‐well plates. Following 4 h of incubation, non‐adherent cells were removed by washing with PBS. Cell adhesion was quantified using a CCK‐8 assay kit (Biosharp), and absorbance was measured with a microplate reader. Adhesion capacity was calculated as the ratio of absorbance of adherent cells to that of total seeded cells.

### 2.20 Immunofluorescence Staining

Cells were seeded on slides and subjected to the respective treatments, followed by three washes with PBS. Cells were fixed with 4% paraformaldehyde and permeabilized with 0.1% Triton X‐100 for 5 min, then blocked with 1% bovine serum albumin (BSA) at room temperature for 1 h. Slides were then incubated overnight at 4°C with primary antibodies against ACAA2 and SIRT5 (detailed antibody information is provided in Table S1). After removal of the primary antibodies and PBS washes, cells were mounted using an anti‐fade reagent containing 4’,6‐diamidino‐2‐phenylindole (DAPI). Images were acquired using an upright fluorescence microscope (Olympus), and quantitative analysis was performed with ImageJ software (version 1.53S).

Paraffin‐embedded mouse kidney sections were deparaffinized, rehydrated, and rinsed with deionized water. Antigen retrieval was performed using microwave heating, followed by blocking for 1 h. Sections were then incubated with primary antibodies against CPT1A, GPX4, HNE, and SIRT5 (detailed antibody information is provided in Table S1). All sections were counterstained with DAPI. Fluorescence images were acquired using a laser scanning confocal microscope.

### 2.21 Immunohistochemical Staining

Paraffin‐embedded mouse kidney tissues were sectioned, deparaffinized, and subjected to antigen retrieval. Sections were incubated overnight at 4°C with primary antibodies, including anti‐αSMA and anti‐Ki67 antibodies (detailed information is provided in Table S1). After removal of the primary antibodies by washing, sections were incubated with secondary antibodies at room temperature for 1 h. This was followed by washing, staining, counterstaining, dehydration, and mounting procedures. The prepared sections were finally observed under an upright microscope (Olympus).

### 2.22 Von Kossa Staining

Paraffin‐embedded mouse kidney tissues were sectioned, deparaffinized, and thoroughly rinsed. The sections were then immersed in silver nitrate staining solution and exposed to ultraviolet light for 10 min. After rinsing, counterstaining, and mounting, the sections were observed under an upright microscope (Olympus).

### 2.23 Periodic Acid‑Schiff (PAS) Staining

Paraffin‐embedded mouse tissues were sectioned, deparaffinized, and rinsed. The sections were stained with periodic acid solution for 15 min, followed by incubation with Schiff's staining solution. After counterstaining and mounting, the sections were observed under an upright microscope (Olympus).

### 2.24 TdT‑Mediated dUTP Nick‑End Labeling (TUNEL) Staining

Apoptosis in kidney tissues was assessed using a TUNEL assay kit (Beyotime). Mouse kidney sections were prepared, deparaffinized, and incubated with proteinase K for 15 min. After thorough washing, the sections were incubated with TUNEL working solution at 37°C for 1 h in the dark. Following additional washing, the samples were immediately observed under a fluorescence microscope (Olympus).

### 2.25 Transmission Electron Microscopy (TEM)

After treatment, cells were sequentially fixed and dehydrated in ethanol and acetone for 15 min each. The samples were then prepared into ultrathin sections (60 nm) and mounted on 200‐mesh copper grids. Finally, the sections were examined using a transmission electron microscope (JEOL, Tokyo, Japan).

### 2.26 Oil Red O Staining

A modified Oil Red O staining kit (Beyotime) was used in this study. Frozen sections were prepared and stored at −20°C. After equilibration to room temperature, the sections were rinsed with 2 mL of staining wash solution, followed by incubation with 1 mL of modified Oil Red O staining solution for 20 min. The sections were then washed again with staining wash solution and further rinsed with PBS. Finally, the samples were observed under an upright microscope (Olympus).

### 2.27 Molecular Docking

The proteins used for docking were ACAA2 (UniProt ID: P42765) and SIRT5 (UniProt ID: Q9NXA8). The molecular structure of MC3138 was obtained from the PubChem database (CID: 155524917). Protein structure models were obtained from the AlphaFold Protein Structure Database (https://alphafold.ebi.ac.uk/). Molecular docking was performed using the HDOCK Server (http://hdock.phys.hust.edu.cn/), and binding energy calculations were conducted using PDBePISA (https://www.ebi.ac.uk/pdbe/pisa/). Finally, interacting sites were visualized using PyMOL 2.4 software.

### 2.28 Molecular Dynamics Simulation

GROMACS 2020.6 was employed to perform a 100 ns molecular dynamics simulation of the protein system. The AMBER99SB‐ILDN force field was used to generate the protein topology. A truncated octahedral TIP3P water box was constructed with a minimum distance of 1.0 nm between the protein and the box boundary, and Na^+^/Cl^−^ ions were added to neutralize the system. Subsequently, a 100 ns production run was carried out under periodic boundary conditions in the NPT ensemble. Long‐range electrostatic interactions were calculated using the particle mesh Ewald (PME) method, with a cutoff distance of 1.0 nm for non‐bonded interactions. The collision frequency was set to 2 ps, the system pressure was maintained at 101.325 kPa, and the integration time step was 2 fs. Trajectories were recorded every 10 ps.

### 2.29 Measurement of Intracellular Fe^2^^+^

Intracellular Fe^2^^+^ levels were visualized using the FerroOrange fluorescent probe (Dojindo). Following the indicated treatments, cells were incubated with 1 µM FerroOrange at 37°C for 30 min. Fluorescence images were then immediately acquired using a confocal laser‐scanning microscope (Olympus). The fluorescence intensity was quantified using ImageJ software (version 1.53s).

### 2.30 Measurement of Oxygen Consumption Rate (OCR)

The OCR was measured using a Seahorse XF24 Extracellular Flux Analyzer (Agilent Technologies, CA, USA). The assay medium was prepared by supplementing phenol red‐free Seahorse XF DMEM medium with pyruvate, glucose, and glutamine. Following the indicated treatments, the culture medium was removed, and the cells were washed and subsequently incubated in assay medium containing BSA‐conjugated palmitate. The cells were then equilibrated for 1 h at 37°C in a non‐CO_2_ incubator. Oligomycin, carbonyl cyanide‐p‐trifluoromethoxyphenylhydrazone (FCCP), and antimycin A were sequentially loaded into the designated injection ports of the sensor cartridge. The hydrated sensor cartridge was then placed onto the cell culture microplate, and OCR measurements were performed using the Seahorse XF24 Extracellular Flux Analyzer. Etomoxir (ETO), a specific inhibitor of CPT1, was used to assess cellular FAO capacity. Etomoxir‐sensitive OCR was calculated as follows: Etomoxir‐sensitive OCR = OCR_Vehicle_—OCR_ETO_.

### 2.31 Statistical Analysis

Statistical analyses were performed using GraphPad Prism software (v9.5.0). Experimental data were derived from at least three independent experiments and are presented as the mean ± standard error of the mean (SEM). Comparisons between two groups were performed using the Student's t‐test, while comparisons among multiple groups were conducted using one‐way analysis of variance (ANOVA) followed by Tukey's multiple comparison test.

## 3 Results

### 3.1 Lipid Metabolic Dysregulation Mediates CaOx‐Induced Oxidative Injury in RTECs

**FIGURE 1:**
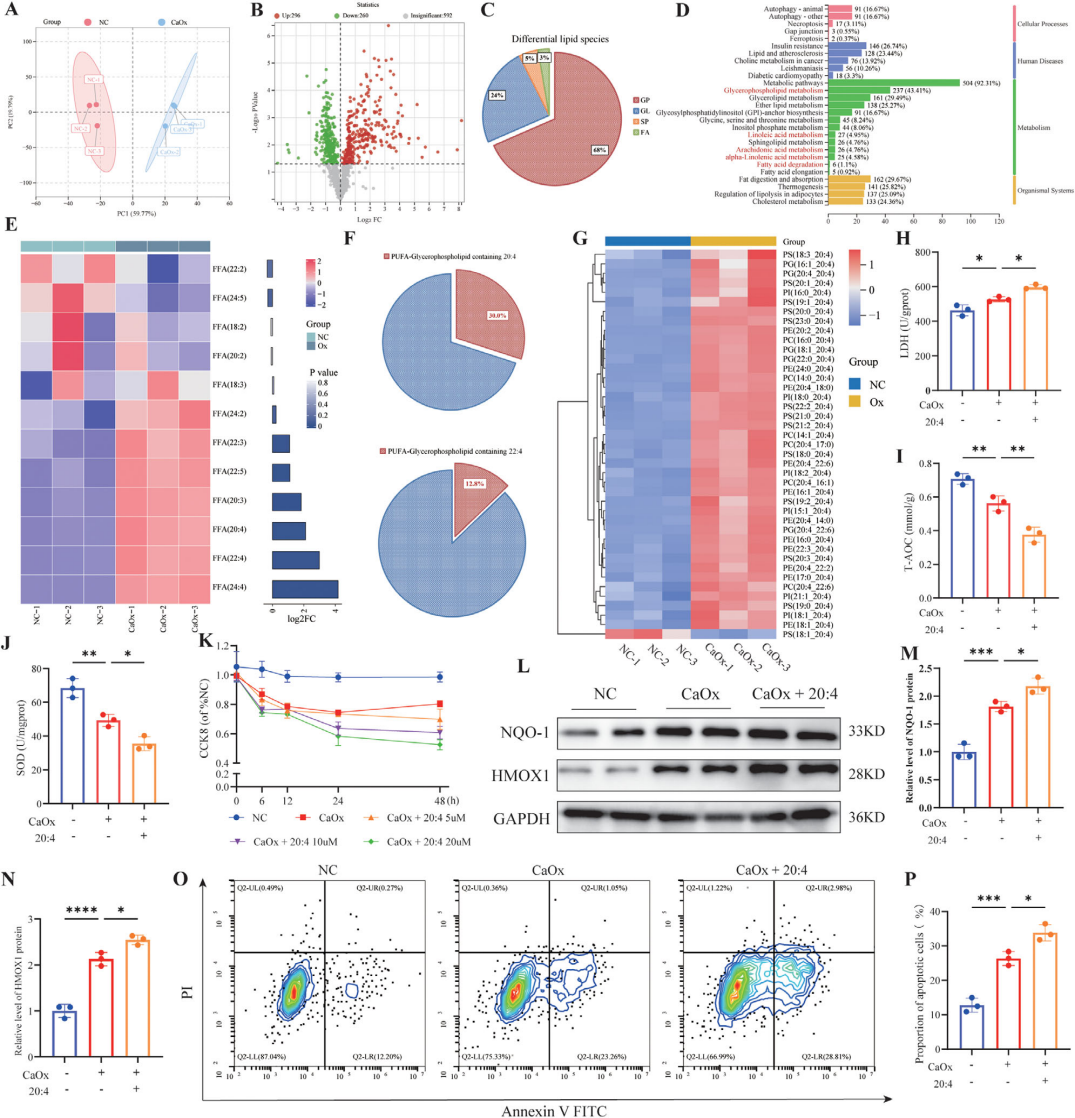
Lipid metabolism dysregulation mediates CaOx‐induced oxidative injury in RTECs. (A) PCA analysis showing the lipid distribution between the model and control groups. (B) Volcano plot depicting differential lipids between the model and control groups. (C) Pie chart of the distribution of differential lipid categories. (D) KEGG enrichment analysis of differential lipids. (E) Distribution of free fatty acids in both groups. (F) Proportion of C20:4 and C22:4 in PUFA‐phospholipids. (G) Heatmap showing the distribution of C20:4‐containing phospholipids. (H) LDH levels, (I) T‐AOC levels, and (J) SOD levels in the three groups. (K) CCK8 assay of HK‐2 cell viability following CaOx and exogenous AA treatment at various concentrations over different time points. (L) Western blot results showing protein levels of NQO‐1 and HMOX1 in the three groups and (M, N) quantitative analysis. (O) Flow cytometry analysis of apoptosis in the three groups and (P) quantitative analysis. The data represent one set of representative images from independent experiments performed at least three times. Results are expressed as mean ± standard error of the mean (SEM). ^*^ *p* < 0.05, ^**^ *p* < 0.01, ^***^ *p* < 0.001, and ^****^ *p* < 0.0001, ns: not significant.

To establish a CaOx crystal‐induced cell injury model, HK‐2 cells were treated with 1.5 mmol/L CaOx for 24 h, which induced moderate cytotoxicity and was used for subsequent analyses (Figure S1A,B). Lipidomic profiling of the CaOx‐induced injury model was performed using LC–MS/MS. The total ion chromatograms of quality control samples exhibited a high degree of overlap, indicating good stability and reproducibility of the mass spectrometry data (Figure S1C). PCA revealed significant alterations in the lipid metabolic profile of RTECs following CaOx treatment (Figure 1A). A total of 556 differential lipid metabolites were identified, including 296 upregulated and 260 downregulated species (Figures 1B and S1D). Among these, glycerophospholipids accounted for the largest proportion (68%), followed by glycerolipids (24%), sphingolipids (5%), and fatty acids (3%) (Figure 1C). KEGG pathway annotation and enrichment analysis based on differential metabolites showed that, in the “Cell Processes” category, pathways such as autophagy, gap junction, and ferroptosis were significantly enriched. In the “Metabolism” category, glycerophospholipid metabolism was prominently enriched. Additionally, several PUFA‐related metabolic pathways, including α‐linolenic acid metabolism, arachidonic acid metabolism, and linoleic acid metabolism, were also enriched (Figure 1D), providing important clues for further investigation. Given the critical regulatory roles of PUFAs in cellular physiology, quantitative analysis of multiple PUFAs was performed. The results demonstrated that several PUFAs were significantly upregulated following CaOx treatment, among which FA (20:4), FA (22:4), and FA (24:4) exhibited the most pronounced increases (Figure 1E). Notably, these elevated PUFAs were largely incorporated into glycerophospholipids. Among the differential PUFA‐containing glycerophospholipids, species containing FA (20:4) accounted for 30.0%, while those containing FA (22:4) accounted for 12.8% (Figure 1F). Quantitative analysis further showed that multiple 20:4‐containing glycerophospholipids were significantly increased in CaOx‐treated cells compared with controls (Figures 1G and S1E).

To further investigate the role of PUFA accumulation in CaOx‐induced cell injury, exogenous FA (20:4) was introduced, and cellular injury was assessed. First, oxidative and antioxidant parameters were evaluated. The results showed that FA (20:4) supplementation further increased LDH release induced by CaOx crystals (Figure 1H). In addition, compared with the CaOx group, the FA (20:4)‐treated group exhibited significantly reduced T‐AOC and SOD levels (Figure 1I,J). Cell viability assays demonstrated that exogenous FA (20:4) decreased HK‐2 cell viability in a time‐ and dose‐dependent manner under CaOx‐induced injury conditions (Figure 1K). Western blot analysis of oxidative stress‐related proteins revealed that CaOx treatment increased the expression of NQO1 and HMOX1 in HK‐2 cells, and this effect was further enhanced by FA (20:4) supplementation (Figure 1L–N). Flow cytometry analysis showed that the proportion of apoptotic cells was significantly higher in the FA (20:4) supplementation group compared with the CaOx group (Figure 1O,P). Taken together, these results indicate that CaOx treatment induces significant lipid metabolic dysregulation in RTECs, and the accumulation of PUFAs exacerbates oxidative injury in these cells.

### 3.2 Abundance of SIRT5 Is Downregulated in the CaOx Kidney Stone Model

**FIGURE 2:**
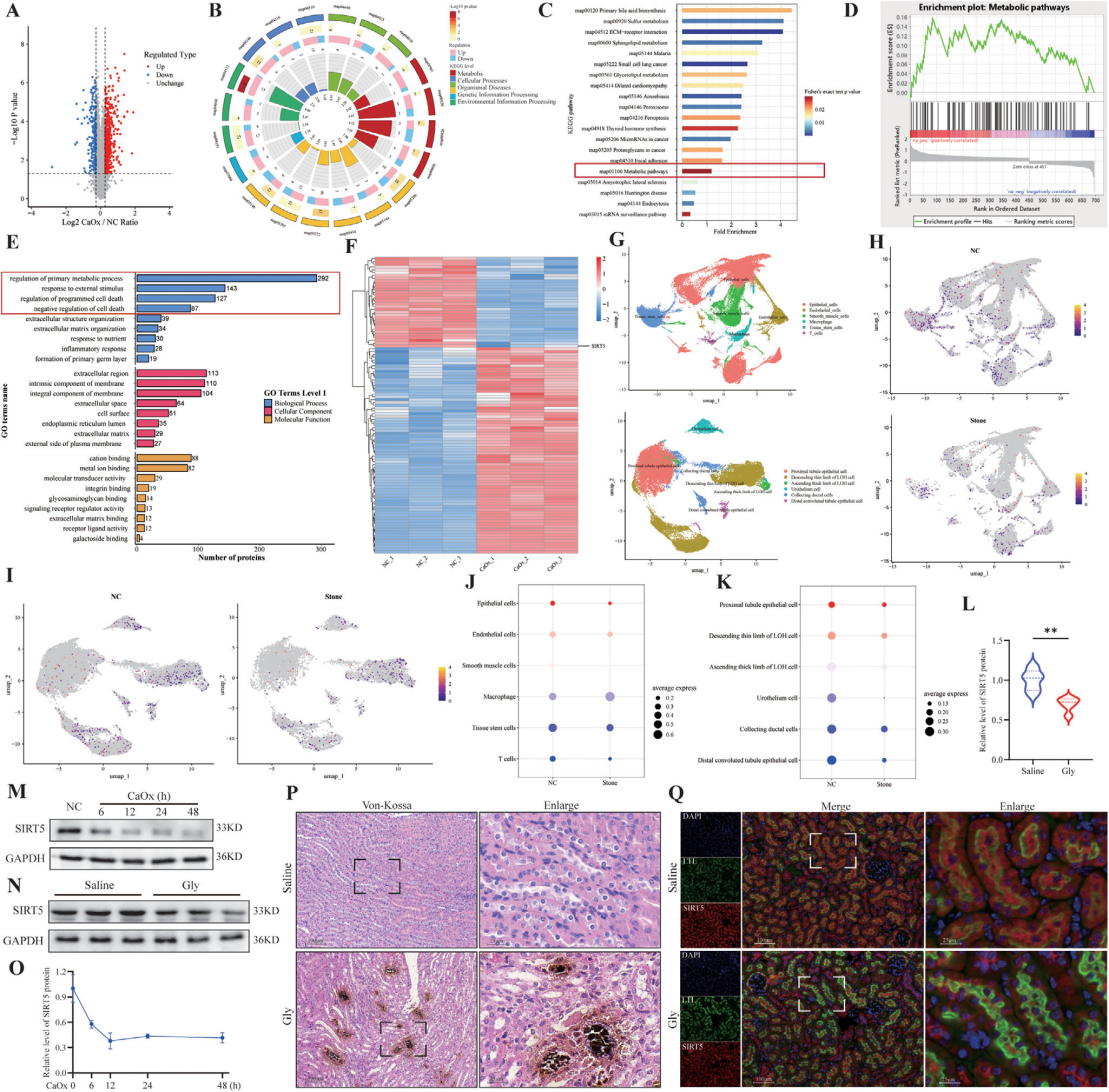
Abundance of SIRT5 is downregulated in the CaOx kidney stone model. (A) The volcano plot shows the differential proteins between the model and control groups. (B) The circular plot illustrates the category distribution of KEGG enrichment for the differential proteins. (C) The bar chart presents the KEGG enrichment analysis of the differential proteins. (D) GSEA analysis of metabolic pathways for the differential proteins in both the model and metabolomic groups. (E) GO enrichment analysis of the differential proteins in both the model and metabolomic groups. (F) The heatmap displays the expression levels of metabolic pathway‐related differential proteins, including SIRT5. (G) Single‐cell data of kidney stone patients and controls reveal clustering of cells and epithelial cell subpopulations. (H) The UMAP plot shows the expression of SIRT5 in the kidney stone patients and controls. (I) The UMAP plot displays SIRT5 expression in epithelial cell subpopulations from both kidney stone patients and controls. (J) The bubble chart illustrates SIRT5 expression across various cell types. (K) The bubble chart shows SIRT5 expression in different cell types within epithelial cell subpopulations. (N) Western blot analysis shows the expression of SIRT5 in mouse kidney tissues from both the model and control groups, (L) along with quantitative analysis. (M) Western blot results show the expression of SIRT5 under different CaOx treatment time gradients, and (O) the corresponding quantitative analysis. (P) Von Kossa staining indicates calcium salt deposition in kidney tissues from both groups. (Q) Immunofluorescence analysis reveals the protein abundance of SIRT5 in the proximal tubular epithelium of mice. The data represent one set of representative images from independent experiments performed at least three times. Results are expressed as mean SEM. ^*^ *p* < 0.05, ^**^ *p* < 0.01, ^***^ *p* < 0.001, and ^****^ *p* < 0.0001, ns: not significant.

To investigate the mechanisms underlying lipid metabolic remodeling in the CaOx crystal–cell injury model, differential proteins induced by CaOx intervention were quantitatively analyzed using 4D‐LFQ proteomics. A total of 696 differential proteins were identified, of which 451 were upregulated and 245 were downregulated (Figure 2A). KEGG and GSEA analyses highlighted the involvement of multiple metabolism‐related pathways in the crystal–cell injury process (Figure 2B–D). GO annotation showed that 292 proteins were associated with the regulation of primary processes. Additionally, pathways related to response to external stimuli, regulation of programmed cell death, and negative regulation of cell death were enriched (Figure 2E). Notably, among the metabolic pathway‐related proteins, SIRT5 was significantly downregulated in the CaOx crystal–cell injury model compared to the NC group (Figure 2F). SIRT5 has been reported to regulate multiple cellular metabolic processes, including lipid metabolism, and to participate in stress response and various forms of programmed cell death through its desuccinylase activity.

Subsequently, SIRT5 expression was analyzed using the human kidney stone single‐nucleus dataset (GSE231568). As shown in Figure 2G, cells were classified into six subpopulations: epithelial cells, endothelial cells, smooth muscle cells, macrophages, tissue stem cells, and T cells. Epithelial cells were further subdivided into proximal tubule cells, descending thin limb LOH cells, ascending thick limb LOH cells, urothelial cells, distal convoluted tubule cells, and collecting duct cells. SIRT5 exhibited a decreasing trend in epithelial cells, endothelial cells, and tissue stem cells (Figure 2H,J). Analysis of epithelial subpopulations revealed that SIRT5 expression was downregulated in multiple renal tubular epithelial cell types, including proximal tubule cells (Figure 2I,K). To further validate SIRT5 expression, an in vitro CaOx time‐course model was established. SIRT5 protein levels gradually decreased from 0 to 6 h of CaOx exposure and then stabilized thereafter (Figure 2M,O). Furthermore, kidney tissues were collected from CaOx crystal‐induced kidney injury mice and NC mice. Von Kossa staining demonstrated significant calcium deposition in the kidneys of the model group (Figure 2P). Western blot analysis confirmed a significant reduction of SIRT5 protein in the model group compared to NC mice (Figure 2L,N). Co‐localization immunofluorescence of SIRT5 with LTL revealed a decreased SIRT5 signal in proximal tubule epithelial cells (Figure 2Q).

### 3.3 SIRT5 Mediates the Desuccinylation of FAO‐Related Enzymes‐ to Regulate‐ Lipid‐ Metabolism

**FIGURE 3:**
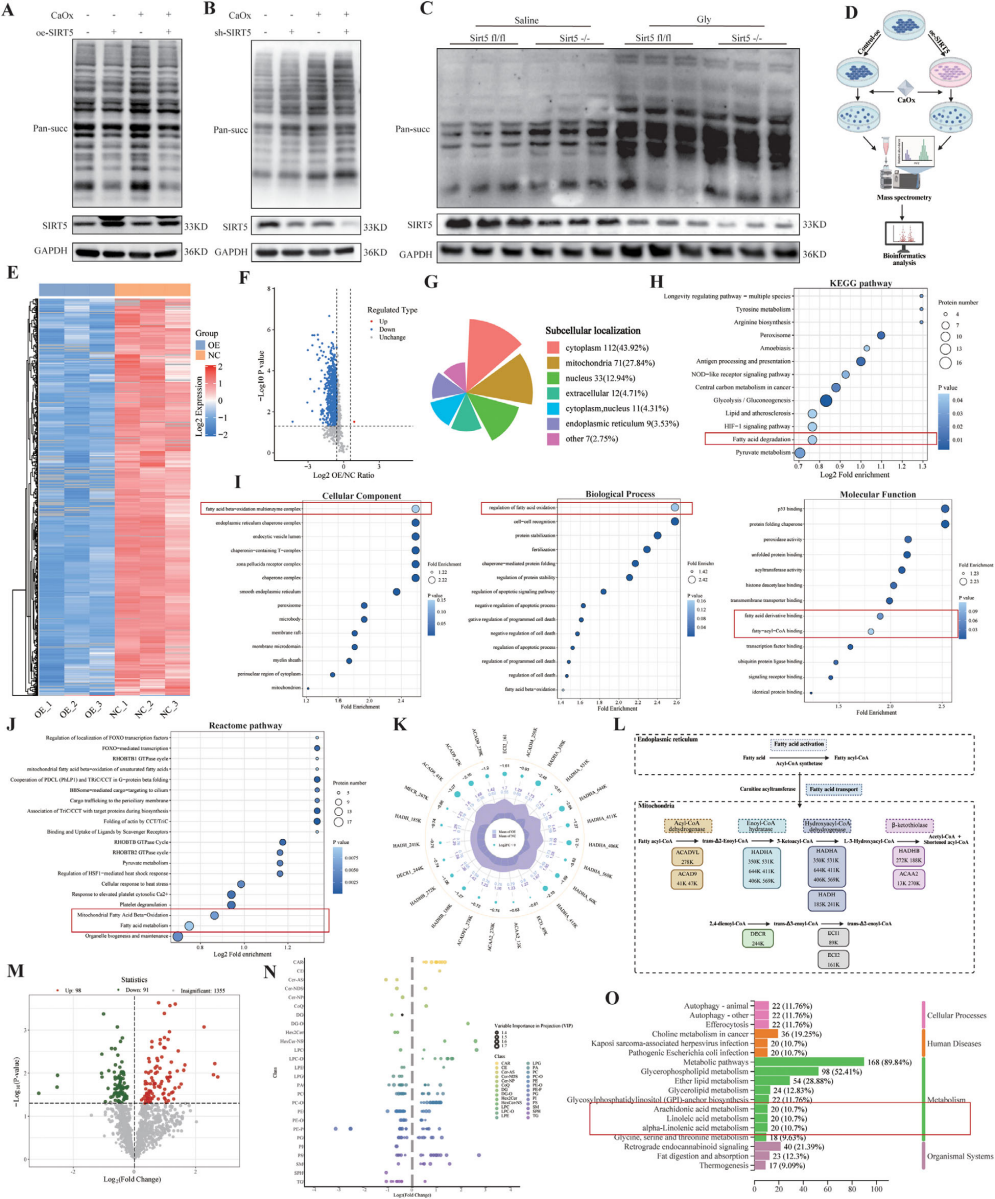
SIRT5 mediates the desuccinylation of FAO‐related enzymes to regulate lipid metabolism. (A) Western blot shows the global protein succinylation after overexpression of SIRT5. (B) Western blot reveals the global protein succinylation following SIRT5 knockdown. (C) Western blot shows the expression of SIRT5 in SIRT5 tubular epithelial cell‐specific knockout mice. (D) SIRT5 knockdown and overexpression cell lines were constructed and subjected to omics analysis. (E) Heatmap displaying the overall protein succinylation modifications. (F) Volcano plot shows the differential succinylation‐modified proteins. (G) Piechart illustrates the subcellular localization of the differential succinylation‐modified proteins. (H) KEGG enrichment analysis of the differential succinylation‐modified proteins. (I) GO enrichment analysis of the differential succinylation‐modified proteins. (J) Reactome enrichment analysis of the differential succinylation‐modified proteins. (K) Radar plot showing the expression of differential succinylation‐modified proteins related to fatty acid metabolism. (L) The steps of FAO involving differential succinylation‐modified proteins. (M) Volcano plot illustrates the differential lipids after overexpression of SIRT5. (N) Forest plot shows the expression of various lipid categories in the two sample groups. (O) KEGG enrichment analysis of the differential lipids. The data represent one set of representative images from independent experiments performed at least three times. Results are expressed as mean SEM. ^*^ *p* < 0.05, ^**^ *p* < 0.01, ^***^ *p* < 0.001, and ^****^ *p* < 0.0001, ns: not significant.

SIRT5 participates in regulating various cellular metabolic pathways through its desuccinylation of mitochondrial metabolic enzymes; therefore, we further investigated the role of its desuccinylase activity in regulation of lipid metabolism. We first generated SIRT5‐overexpressing (oe‐SIRT5) and knockdown (sh‐SIRT5) HK‐2 cell lines, and validated transfection efficiency by Western blot (Figure S2A–D). Western blot analysis using a pan‐succinyllysine antibody revealed that CaOx treatment increased global protein succinylation in HK‐2 cells, while SIRT5 overexpression partially reduced succinylation levels and SIRT5 knockdown exhibited the opposite effect (Figure 3A,B). We then generated tubular epithelial cell–specific SIRT5 knockout mice and established an in vivo CaOx kidney stone model. Genotyping of mice was confirmed by DNA agarose gel electrophoresis (Figure S2E), and knockout efficiency was validated by Western blot (Figure S2F,G). Similarly, in the in vivo CaOx nephrolithiasis model, tubular‐specific deletion of SIRT5 elevated overall renal protein succinylation (Figure 3C).

To characterize SIRT5‐mediated desuccinylation, LC‐MS/MS was performed on WT and oe‐SIRT5 HK‐2 cells (Figure 3D). Peptides predominantly ranged from 7 to 20 amino acids, meeting analytical criteria (Figure S2H), and the data demonstrated high reproducibility and stability (Figure S2I,J). SIRT5 overexpression reduced overall succinylation, with 656 lysine sites across 255 proteins showing significant decreases in the oe‐SIRT5 group (Figure 3E,F). Subcellular localization analysis indicated that these differentially succinylated proteins were mainly cytosolic (43.92%) and mitochondrial (27.84%) (Figure 3G). KEGG pathway enrichment analysis revealed significant involvement in fatty acid degradation, with nine FAO‐related proteins exhibiting altered succinylation upon SIRT5 overexpression (Figure 3H). In addition, GO analysis based on differentially succinylated proteins provided annotation of cellular components, molecular functions, and biological processes. Within the cellular component category, significant enrichment was observed for the FAO multienzyme complex (7 proteins). In the biological process category, a total of 26 proteins were found to be associated with the regulation of FAO, indicating significant enrichment. The molecular function category revealed enrichment for fatty acid derivative binding and fatty‐acyl‐CoA binding (Figure 3I). Similarly, Reactome pathway enrichment analysis indicated significant enrichment of mitochondrial FAO and fatty acid metabolism (Figure 3J). Based on the enrichment analysis results, we focused on FAO‐related proteins. Among these, ACADVL (278K) and ACAD9 (47 and 41K) are involved in acyl‐CoA dehydrogenation; HADHA (350K, 531K, 644K, 411K, 406 and 569K) participates in enoyl‐CoA hydration and hydroxyacyl‐CoA dehydrogenation; and HADHB (272 and 188K) and ACAA2 (13 and 270K) are associated with β‐ketothiolase activity. Additionally, DECR1 (244K) is involved in the reduction of unsaturated fatty acids, whereas ECI1 (89K) and ECI2 (161K) are involved in the isomerization of unsaturated fatty acids (Figure 3K,L).

We further performed broad‐target lipidomics analysis on WT and oe‐SIRT5 HK‐2 cells under CaOx treatment. Following quality control, PCA demonstrated that SIRT5 overexpression reprogrammed cellular lipid metabolism (Figure S2K,L). A total of 189 differential lipid species were identified, including 98 upregulated and 91 downregulated (Figure 3M). Notably, multiple acylcarnitines (CAR), triglycerides (TG), phosphatidylethanolamines (PE), phosphatidylserines (PS), phosphatidylcholines (PC), and phosphatidylglycerols (PG) were significantly altered in oe‐SIRT5 cells (Figure 3N). KEGG analysis of differential lipids revealed strong enrichment in glycerophospholipid metabolism, alpha‐linolenic acid metabolism, arachidonic acid metabolism, and linoleic acid metabolism (Figure 3O).

### 3.4 SIRT5 Deficiency‐Mediated FAO Dysfunction Leads to Mitochondrial Oxidative Injury in the CaOx Crystal‐Induced Cell Injury Model

**FIGURE 4:**
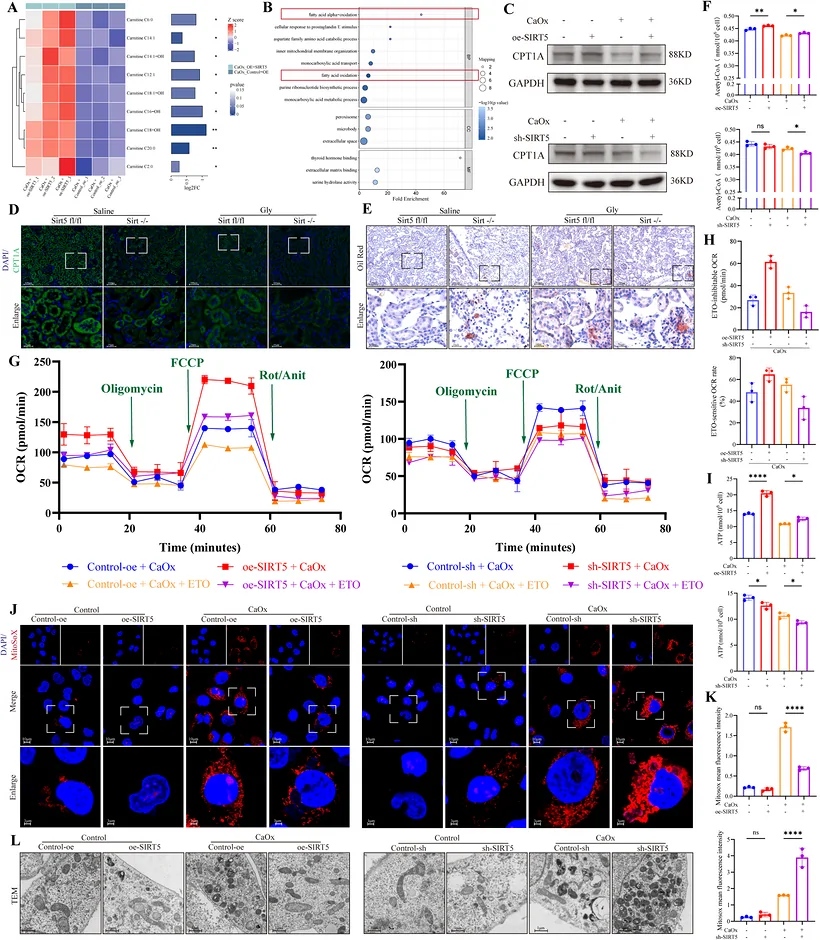
SIRT5 deficiency‐mediated FAO dysfunction leads to mitochondrial oxidative injury in the CaOx crystal‐induced cell injury model. (A) A heatmap illustrates the differential expression of acylcarnitines following SIRT5 overexpression. (B) GO enrichment analysis was performed on the differentially expressed proteins after SIRT5 overexpression. (C) Western blot analysis shows CPT1A expression following SIRT5 overexpression and knockdown, along with its quantitative analysis. (D) Immunofluorescence staining demonstrates CPT1A expression in kidney tissue samples from four groups of mice. (E) Oil Red O staining was performed on kidney tissues from the four groups. (F) Acetyl‐CoA levels were measured in samples from each group. (G) Seahorse measured OCR profile of Control‐oe, oe‐SIRT5, Control‐sh, sh‐SIRT5 cells with or without etomoxir treatment, followed by injections of mitochondrial respiration inhibitors oligomycin, FCCP, Rot/Anit indicated by arrows. (H) The bar graph shows the ETO‐inhibitable OCR and ETO‐sensitive OCR in four groups of cells. (I) ATP levels were measured in samples from each group. (J) MitoSOX probe was used to detect mitochondrial ROS levels in cells from each group, with (K) corresponding quantitative analysis. (L) TEM reveals morphological changes in mitochondria in cells from each group. The data represent one set of representative images from independent experiments performed at least three times. Results are expressed as mean SEM. ^*^ *p* < 0.05, ^**^ *p* < 0.01, ^***^ *p* < 0.001, and ^****^ *p* < 0.0001, ns: not significant.

To further investigate the regulatory effect of SIRT5 on mitochondrial FAO in HK‐2 cells, SMPDB enrichment analysis was performed based on differential lipid metabolites between the oe‐SIRT5 and NC groups. This analysis revealed that O‐acetylcarnitine, a metabolite associated with beta‐oxidation of very long chain fatty acids, was significantly increased in the SIRT5 overexpression group (Figure S3A,B). O‐acylcarnitines are intermediate metabolites involved in fatty acid transport during FAO, and multiple acylcarnitines were significantly elevated in the oe‐SIRT5 group (Figure 4A), suggesting a positive regulatory role of SIRT5 in FAO. Subsequently, 4D‐LFQ proteomics was employed to identify differential proteins in SIRT5 overexpressing HK‐2 cells (Figure S3C,D). Upregulated GO enrichment analyses highlighted pathways including FAO and fatty acid metabolic processes, with several associated proteins significantly increased in the oe‐SIRT5 group (Figure 4B). Among these, CPT1A, a key regulator of FAO, exhibited significant changes. Its protein level was markedly reduced following CaOx treatment, whereas SIRT5 overexpression partially restored CPT1A expression, and SIRT5 knockdown produced the opposite effect (Figure 4C and Figure S3E). Similarly, in the CaOx nephrolithiasis model, tubular‐specific deletion of SIRT5 decreased CPT1A expression in renal tissues (Figure 4D and Figure S3F). SIRT5 tubular‐specific knockout also exacerbated lipid accumulation in mouse renal tissue (Figure 4E). Acetyl‐CoA, a key product of the FAO reaction, was significantly increased upon SIRT5 overexpression and decreased upon SIRT5 knockdown (Figure 4F). To directly assess cellular FAO activity, HK‐2 cells were treated with ETO, an inhibitor of CPT1, and the OCR was measured using a Seahorse extracellular flux analyzer. Under CaOx exposure, the overall OCR was significantly higher in the oe‐SIRT5 group than in the control‐oe group, and ETO treatment induced a pronounced reduction in OCR. In contrast, SIRT5 knockdown markedly decreased the overall OCR and attenuated the cellular response to ETO (Figure 4G). Quantitative analysis further showed that both ETO‐inhibitable OCR and the ETO‐sensitive OCR rate were increased following SIRT5 overexpression but decreased following SIRT5 knockdown (Figure 4H). These findings indicate that SIRT5 overexpression enhances FAO activity in HK‐2 cells under CaOx stress, whereas SIRT5 knockdown impairs FAO.

Impairment of mitochondrial FAO may further lead to mitochondrial dysfunction and oxidative injury. Accordingly, multiple mitochondrial damage indicators were assessed. As the core organelle for energy production, mitochondrial ATP production was increased by SIRT5 overexpression, whereas the sh‐SIRT5 group showed reduced ATP production (Figure 4I). Flow cytometry analysis revealed that ROS levels were significantly reduced in the oe‐SIRT5 + CaOx group compared with the control‐oe + CaOx group, whereas SIRT5 knockdown produced the opposite effect (Figure S3G,H). JC‐1 staining showed that mitochondrial membrane potential was further reduced following SIRT5 knockdown compared with the model group, while SIRT5 overexpression partially restored it (Figure S3I). MitoSOX probe was used to detect mitochondrial ROS levels; SIRT5 overexpression significantly reduced mitochondrial ROS levels compared with the control‐oe + CaOx group, whereas SIRT5 knockdown aggravated mitochondrial ROS accumulation (Figure 4J,K). Furthermore, SIRT5 overexpression significantly alleviated CaOx‐induced mitochondrial rounding, swelling, and fragmentation in HK‐2 cells, whereas SIRT5 knockdown exacerbated mitochondrial damage (Figure 4L).

### 3.5 SIRT5 Deficiency Exacerbates Ferroptosis and Crystal Adhesion in a CaOx Crystal–Induced Cell Injury Model

Previous studies have demonstrated that ferroptosis in renal tubular epithelial cells plays an important role in CaOx stone formation, whereas FAO may influence cellular susceptibility to ferroptosis by promoting PUFA catabolism. Therefore, we treated HK‐2 cells were with fenofibrate, a PPARα agonist, to investigate the relationship between FAO and ferroptosis in renal tubular epithelial cells. Western blot analysis showed that CaOx exposure markedly reduced the levels of the anti‐ferroptotic proteins xCT, GPX4, and FTH, whereas fenofibrate treatment partially restored their expression. In addition, fenofibrate attenuated the CaOx‐induced upregulation of ACSL4, a key pro‐ferroptotic enzyme (Figure S4A,B). Lipid peroxidation was further evaluated using the C11 BODIPY 581/591 probe. CaOx exposure significantly increased lipid peroxidation in HK‐2 cells, whereas lipid peroxidation was markedly reduced in the CaOx + fenofibrate group compared with the CaOx‐treated group (Figure S4C). Collectively, these findings support the involvement of impaired FAO in CaOx‐induced ferroptosis in renal tubular epithelial cells.

**FIGURE 5:**
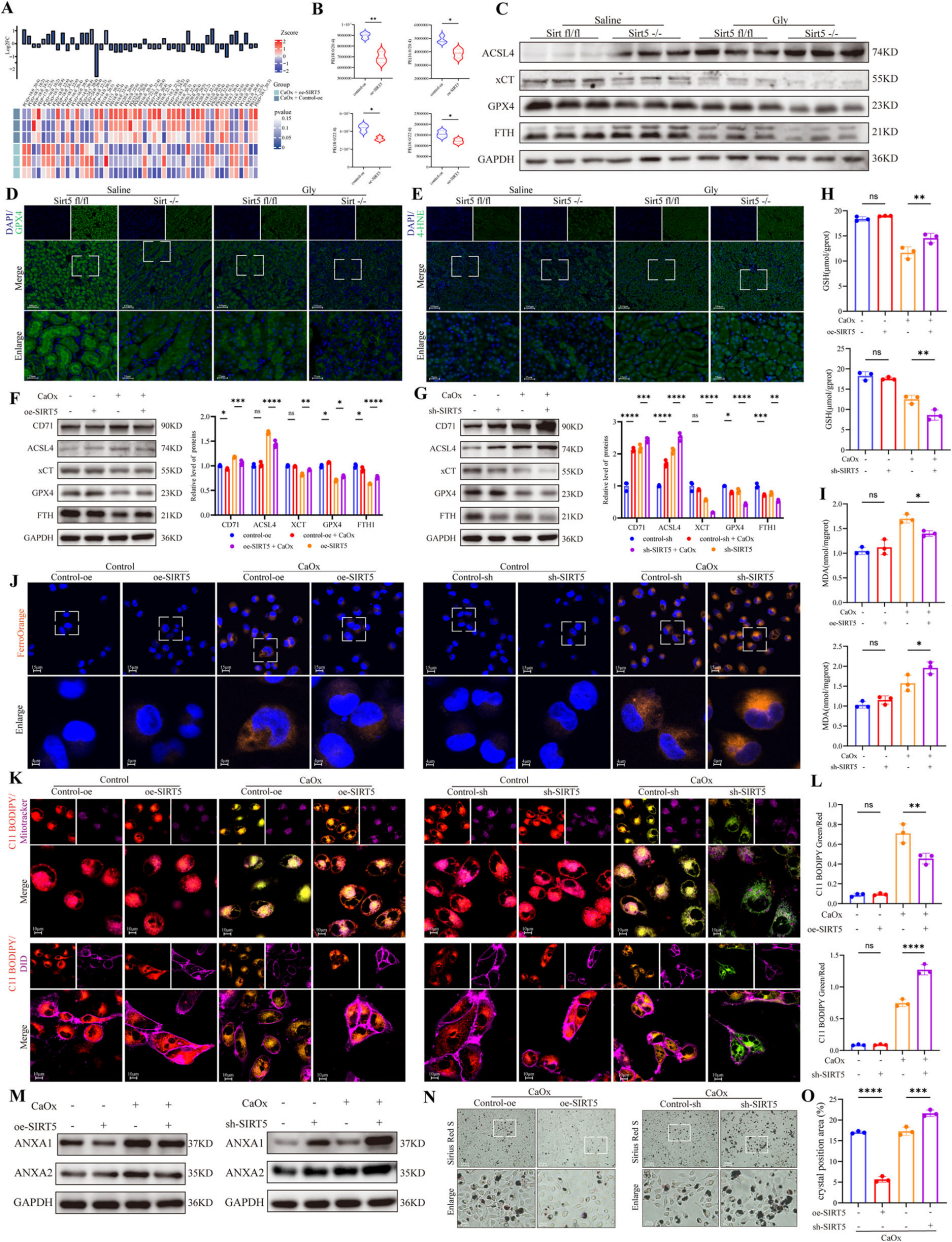
SIRT5 deficiency exacerbates ferroptosis and crystal adhesion in a CaOx crystal–induced cell injury model. (A) The heatmap shows the differential expression of polyunsaturated phospholipids following overexpression of SIRT5. (B) The expression levels of PE (18:0/20:4), PE (16:0/20:4), PE (16:0/22:4), and PE (18:0/22:4) were measured following SIRT5 overexpression. (C) Western blot analysis reveals the expression of ACSL4, xCT, GPX4, and FTH in kidney tissues from SIRT5 tubular epithelial cell‐specific knockout mice. (D) Immunofluorescence staining demonstrates the expression of GPX4 in kidney tissues from SIRT5 tubular epithelial cell‐specific knockout mice. (E) Immunofluorescence staining shows the expression of 4‐HNE in kidney tissues from SIRT5 tubular epithelial cell‐specific knockout mice. (F) Western blot analysis shows the expression levels of CD71, ACSL4, xCT, GPX4, and FTH in cells after overexpressing SIRT5. (G) Western blot analysis shows the expression levels of CD71, ACSL4, xCT, GPX4, and FTH in cells following SIRT5 knockdown. (H) The GSH levels in the samples from each group. (I) The MDA levels in the samples from each group. (J) Intracellular Fe^2^^+^ levels were assessed using the FerroOrange probe following SIRT5 overexpression or knockdown. (K) The lipid peroxidation was assessed by co‐staining C11 BODIPY with Mitotracker and DID in cells after overexpression or knockdown of SIRT5. (L) Quantitative analysis of C11 BODIPY staining in cells from each group. (M) Western blot analysis shows the expression of the adhesion factors ANXA1 and ANXA2 in cells from each group. (N) Representative images show the adhesion of CaOx crystals to HK‐2 cells. (O) Quantitative analysis of CaOx crystal adhesion to HK‐2 cells in each group. The data represent one set of representative images from independent experiments performed at least three times. Results are expressed as mean SEM. ^*^ *p* < 0.05, ^**^ *p* < 0.01, ^***^ *p* < 0.001, and ^****^ *p* < 0.0001, ns: not significant.

SIRT5 deficiency–induced impairment of mitochondrial FAO may promote the incorporation of PUFAs into phospholipids, rendering them susceptible substrates for lipid peroxidation and thereby increasing the sensitivity of HK‐2 cells to ferroptosis. LC–MS/MS analysis revealed that SIRT5 overexpression reduced the levels of multiple PUFA‐containing glycerophospholipids (Figure 5A). Notably, PE (18:0/20:4), PE (16:0/20:4), PE (16:0/22:4), and PE (18:0/22:4), which are recognized pro‐ferroptotic signals, were significantly decreased upon SIRT5 overexpression (Figure 5B). We next evaluated ferroptosis‐related markers in an in vivo CaOx nephrolithiasis model. Compared with the model group, renal tubular–specific deletion of SIRT5 further decreased the levels of anti‐ferroptotic proteins xCT, GPX4, and FTH, while increasing the expression of the pro‐ferroptotic protein ACSL4 (Figures 5C and S4D). Immunofluorescence staining of GPX4 in renal tissues yielded consistent results (Figures 5D and S4E). In addition, SIRT5 conditional knockout further elevated the accumulation of 4‐HNE in renal tissues compared with the model group (Figures 5E and S4F). In vitro validation showed that SIRT5 overexpression partially attenuated CaOx‐induced upregulation of ACSL4 and CD71 in HK‐2 cells, while restoring, to some extent, the levels of xCT, GPX4, and FTH (Figure 5F). In contrast, SIRT5 knockdown produced the opposite effects (Figure 5G). The level of GSH, a cofactor of GPX4, was partially restored upon SIRT5 overexpression but further depleted following SIRT5 knockdown (Figure 5H). Consistently, MDA levels were reduced in the oe‐SIRT5 + CaOx group compared with the control‐oe + CaOx group, whereas SIRT5 knockdown exacerbated MDA accumulation (Figure 5I). Intracellular Fe^2^^+^ levels were assessed using the FerroOrange fluorescent probe. The results showed that SIRT5 overexpression partially attenuated the CaOx‐induced increase in intracellular Fe^2^^+^ levels, whereas SIRT5 knockdown further exacerbated Fe^2^^+^ accumulation (Figure 5J and Figure S4G). As phospholipids are essential components of cellular membranes, the accumulated unsaturated phospholipids may not be confined to the mitochondrial membrane but may also be enriched in the plasma membrane. The C11 BODIPY probe was further used to detect oxidized lipids and assess their subcellular localization. Co‐staining with MitoTracker revealed that lipid peroxidation within the mitochondrial region was partially reduced upon SIRT5 overexpression and increased following SIRT5 knockdown. Using the DiD probe to label the plasma membrane, we found that oxidized lipid levels within the plasma membrane region were significantly decreased in the oe‐SIRT5 group compared with the model group, whereas an increasing trend was observed in the sh‐SIRT5 group (Figure 5K,L). Oxidative damage to the plasma membrane may provide binding sites for crystal adhesion. Analysis of key adhesion molecules ANXA1 and ANXA2 showed that CaOx stimulation markedly upregulated their expression, while SIRT5 overexpression partially reduced their abundance, and SIRT5 knockdown exerted the opposite effect (Figure 5M and S4H,I). Moreover, analysis of cell adhesion capacity revealed that SIRT5 overexpression alleviated the CaOx‐induced increase in cell adhesion, whereas SIRT5 knockdown further enhanced the adhesion capacity of HK‐2 cells (Figure S4J). Sirius Red S staining was used to visualize CaOx crystals and assess cell‐crystal adhesion. The results indicated that SIRT5 overexpression alleviated the adhesion of HK‐2 cells to CaOx crystals, whereas SIRT5 knockdown exacerbated the cell‐crystal adhesion phenomenon (Figure 5N,O).

### 3.6 SIRT5 Deficiency Exacerbates Crystal Deposition and CaOx‐Induced Kidney Injury

**FIGURE 6:**
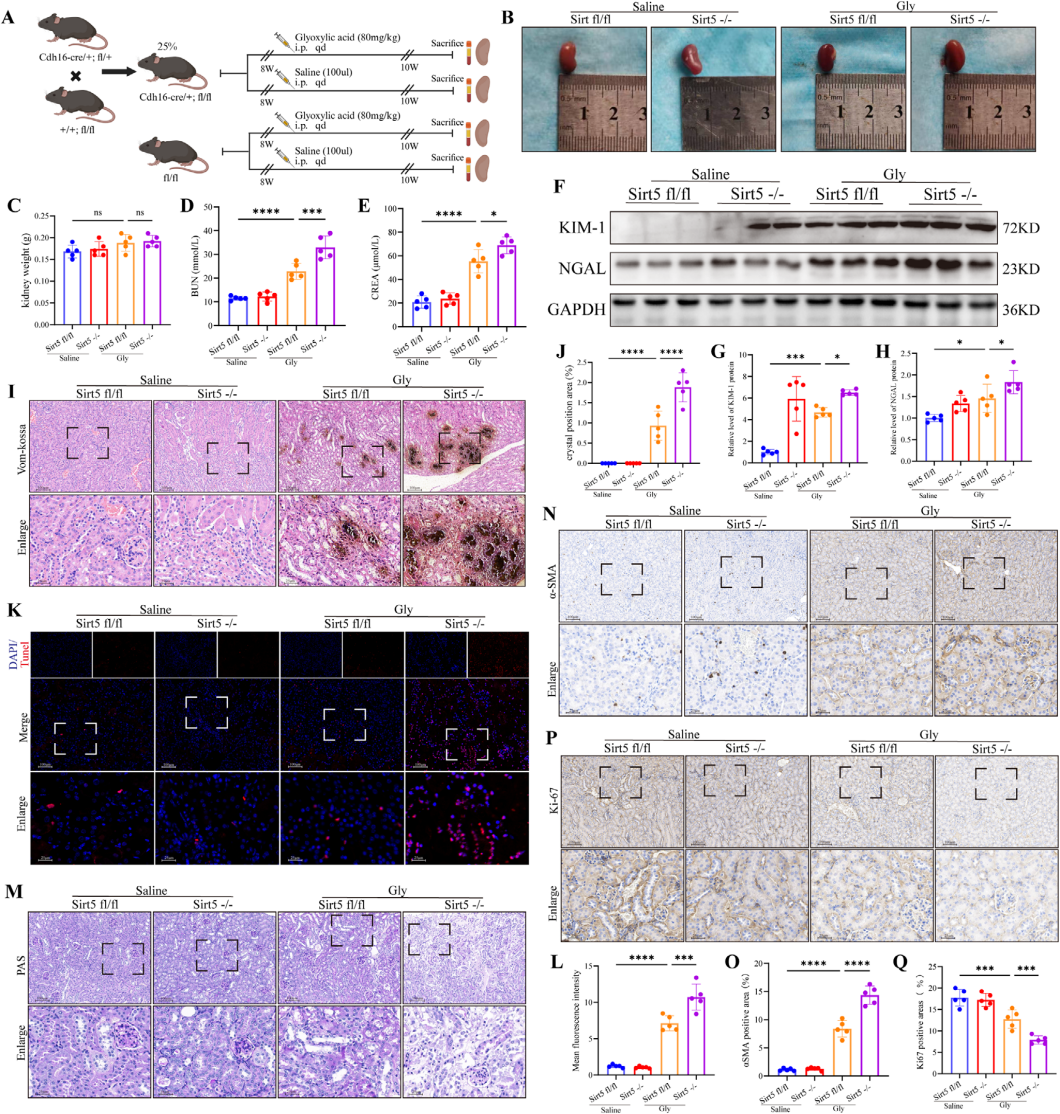
SIRT5 Deficiency Exacerbates Crystal Deposition and CaOx‐Induced Kidney Injury. (A) Schematic diagram of the animal experimental workflow. (B) Representative images of kidneys from the four groups of mice. (C) Bar graph shows the kidney weights of mice in each group. (D) Serum BUN levels were measured in the four groups of mice. (E) Serum CREA levels were measured in the four groups of mice. (F) Western blot analysis shows the expression of KIM‐1 and NGAL in kidney tissues from the four groups, with (G, H) corresponding quantitative analysis. (I) Von Kossa staining illustrates calcium salt deposition in the kidneys of the four groups, with (J) quantitative analysis. (K) Representative images of TUNEL staining in kidney tissues from the four groups are shown, with (L) quantitative analysis. (M) Periodic acid–Schiff (PAS) staining reveals the extent of kidney injury in the four groups. (N) Immunohistochemical staining of αSMA in kidney tissues from the four groups is shown, with (O) corresponding quantitative analysis. (P) Immunohistochemical staining of Ki‐67 in kidney tissues from the four groups is presented, with (Q) corresponding quantitative analysis. The data represent one set of representative images from independent experiments performed at least three times. Results are expressed as mean SEM. ^*^ *p* < 0.05, ^**^ *p* < 0.01, ^***^ *p* < 0.001, and ^****^ *p* < 0.0001, ns: not significant.

Crystal adhesion to renal tubular epithelial cells is a key initiating step in kidney stone formation. The researchers further validated the regulatory role of SIRT5 in crystal deposition and renal injury using mice with renal tubule‐specific SIRT5 knockout (Figure 6A). As shown in Figure 6B,C, neither Gly intervention nor SIRT5 knockout significantly affected kidney size. Renal function assessment revealed that BUN and serum creatinine levels were significantly elevated in the model group compared to controls, and SIRT5 knockout further aggravated renal dysfunction (Figure 6D,E). Consistently, Western blot analysis showed that SIRT5 knockout modestly increased the expression of renal injury markers KIM‐1 and NGAL (Figure 6F–H). Notably, even under saline treatment, renal tubular SIRT5 deletion resulted in an upward trend in both KIM‐1 and NGAL expression, with a more pronounced increase in KIM‐1 (Figure 6F–H). As an early marker of tubular injury, KIM‐1 is sensitive to localized and low‐grade tubular epithelial damage. We therefore speculate that loss of SIRT5 in RTECs compromises tubular metabolic reserve, predisposing these cells to a state of subclinical or early metabolic stress. Moreover, KIM‐1 also functions as a phosphatidylserine‐recognizing receptor. Thus, its preferential upregulation may additionally represent an adaptive response to mild membrane injury caused by SIRT5 deficiency. Von Kossa staining demonstrated that Gly induced calcium salt deposition, which were further exacerbated by SIRT5 deficiency (Figure 6I,J). TUNEL staining indicated that renal apoptosis was more pronounced in SIRT5‐deficient mice than in the model group (Figure 6K,L). Moreover, CaOx crystal deposition caused tubular damage, dilation, and basement membrane exposure, which were aggravated upon SIRT5 knockdown (Figure 6M). Immunohistochemical analysis of αSMA revealed that SIRT5 deficiency further intensified CaOx‐induced renal fibrosis (Figure 6N,O). Additionally, assessment of the proliferation marker Ki67 showed that SIRT5 knockdown partially worsened CaOx‐induced reductions in cellular viability (Figure 6P,Q).

### 3.7 SIRT5‐Mediated Desuccinylation of ACAA2 at K13 Residue Regulates Its Enzymatic Activity

**FIGURE 7:**
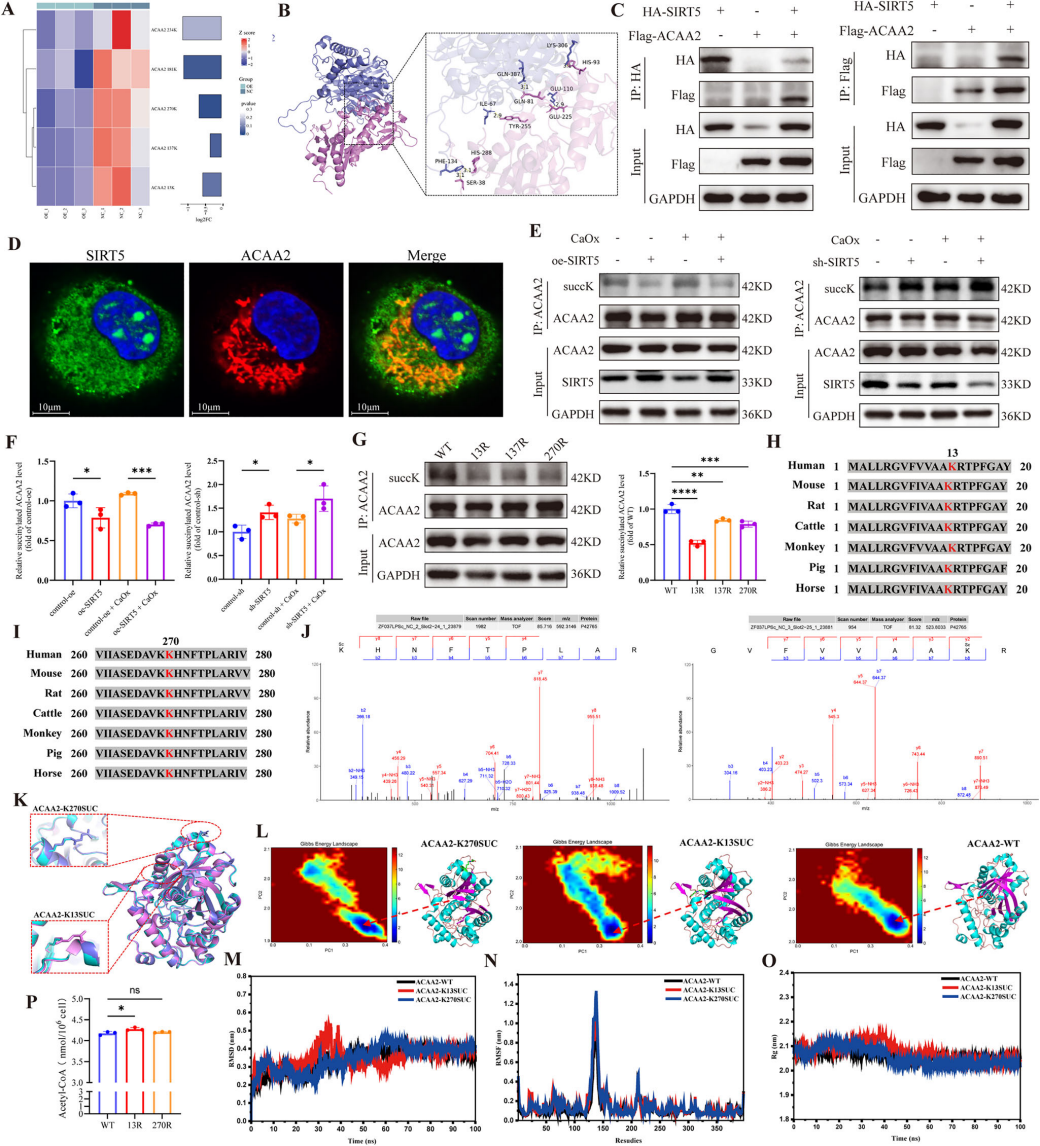
SIRT5‐mediated desuccinylation of ACAA2 at K13 residue regulates its enzymatic activity. (A) The heatmap displays the changes in site‐specific succinylation of ACAA2 following SIRT5 overexpression. (B) Molecular docking analysis shows the binding interaction between SIRT5 and ACAA2. (C) CoIP experiments demonstrate the interaction between SIRT5 and ACAA2. (D) Immunofluorescence co‐staining shows the expression patterns of SIRT5 and ACAA2 in the cells. (E, F) CoIP experiments reveal the succinylation levels of ACAA2 after modulation of SIRT5, and the corresponding quantitative analysis. (G) CoIP experiments demonstrate changes in ACAA2 succinylation levels following K13R, K137R, and K270R point mutations, with the quantitative analysis. (H) K13 (marked in red) is evolutionarily conserved in ACAA2. (I) K270 (marked in red) is evolutionarily conserved in ACAA2. (J) MS spectrum of the succinylated ACAA2 K13 and K270 site. (K) The structural comparison of ACAA2 succinylation at K13 and K270 is shown. (L) Molecular dynamics simulations were performed to analyze the succinylation of ACAA2 at K13 and K270. (M) The image shows the RMSD of ACAA2 protein after succinylation at K13 and K270. (N) The image shows the RMSF of ACAA2 protein after succinylation at K13 and K270. (O) The images show the radius of Rg of ACAA2 protein after succinylation at K13 and K270. (P) The intracellular acetyl‐CoA levels were measured following the point mutations of ACAA2. The data represent one set of representative images from independent experiments performed at least three times. Results are expressed as mean SEM. ^*^ *p* < 0.05, ^**^ *p* < 0.01, ^***^ *p* < 0.001, and ^****^ *p* < 0.0001, ns: not significant.

Among the FAO‐related differentially succinylated proteins, ACAA2, which catalyzes the final step of the β‐ketothiolase reaction and is a key rate‐limiting enzyme, exhibited decreased succinylation levels at multiple sites following SIRT5 overexpression (Figure 7A), prompting further investigation. Molecular docking analysis revealed a stable interaction between SIRT5 and ACAA2, suggesting their potential interaction (Figure 7B). Subsequently, HK‐2 cells were co‐transfected with Flag‐ and HA‐tagged ACAA2 and SIRT5, respectively, and Co‐IP experiments further validated the binding between SIRT5 and ACAA2 (Figure 7C). Immunofluorescence co‐staining further demonstrated the colocalization of SIRT5 and ACAA2 (Figure 7D). CaOx treatment over a time gradient led to a progressive decrease in SIRT5 expression, whereas ACAA2 expression levels showed no significant change (Figure S5A), indicating that SIRT5 regulates the succinylation level of ACAA2 without affecting its total protein expression. Subsequent Co‐IP assays were performed to assess the succinylation status of ACAA2. CaOx treatment significantly increased ACAA2 succinylation, whereas SIRT5 overexpression partially reduced its succinylation level. In contrast, SIRT5 knockdown further enhanced ACAA2 succinylation (Figure 7E,F). Based on the LC‐MS/MS results, we constructed mutant plasmids of ACAA2 at residues K13, K137, and K270, substituting lysine (K) with arginine (R) to mimic the desuccinylated state, and transfected these mutants along WT ACAA2 into HK‐2 cells (Figure S5B). As shown in Figure 7G, K13R and K270R mutations resulted in the most pronounced reduction in ACAA2 succinylation levels. Notably, the K13 and K270 residues are also highly conserved across multiple species, including humans, mice, rats, and cattle (Figure 7H,I). The tandem MS spectra confirmed lysine succinylation at the K13 and K270 residues of ACAA2 (Figure 7J). Molecular dynamics simulations were then conducted to evaluate the impact of succinylation at K13 and K270 on ACAA2 structural stability (Figure 7K). Succinylation at both sites induced conformational alterations in the protein (Figure 7L). Compared with WT ACAA2, succinylation at either site increased RMSD fluctuations, prolonged the time required to reach stability, and resulted in higher RMSD values at equilibrium (Figure 7M). Residue flexibility, assessed by RMSF, was also elevated following succinylation. The effect was more pronounced at K13, which significantly increased RMSF values in the N‐terminal region, with most residues exhibiting enhanced flexibility (Figure 7N). In addition, both K13 mutation and K270 succinylation extended the fluctuation duration of the radius of gyration (Rg) relative to WT ACAA2 (Figure 7O). Finally, intracellular acetyl‐CoA levels were measured in HK‐2 cells expressing WT or mutant ACAA2. The K13R mutation significantly increased acetyl‐CoA levels (Figure 7P). Taken together, these results demonstrate that SIRT5 primarily regulates ACAA2 enzymatic activity through desuccinylation at the K13 residue.

### 3.8 Enhanced ACAA2 Alleviates CaOx Crystal–Induced Oxidative Stress and Ferroptosis in RTECs

To further investigate the role of ACAA2 in CaOx crystal–induced cellular injury, HK‐2 cells overexpressing ACAA2 were generated via plasmid transfection, and transfection efficiency was confirmed by Western blot analysis. Assessment of oxidative stress–related proteins showed that ACAA2 overexpression partially attenuated the CaOx‐induced upregulation of NQO‐1 and HMOX1. However, supplementation with exogenous FA 20:4 restored the expression levels of these proteins (Figure S5C). Cell viability was evaluated using the CCK‐8 assay, which demonstrated that ACAA2 overexpression significantly improved cell viability under CaOx treatment, whereas the addition of FA 20:4 reduced cell viability (Figure S5D). T‐AOC and SOD activity were measured as indicators of cellular antioxidant capacity. Compared with the Control‐oe + CaOx group, the oe‐ACAA2 + CaOx group exhibited significantly elevated T‐AOC and SOD levels, while FA 20:4 supplementation reversed these effects (Figure S5E,F). Furthermore, ACAA2 overexpression partially reduced intracellular LDH accumulation induced by CaOx crystals, whereas FA 20:4 treatment increased LDH levels (Figure S5G). To further determine the role of ACAA2 in SIRT5 deficiency–induced ferroptosis, ACAA2 was ectopically expressed by plasmid transfection in SIRT5‐knockdown HK‐2 cells. Western blot analysis showed that, under CaOx stress, the expression levels of the anti‐ferroptotic proteins xCT and GPX4 were higher in the sh‐SIRT5 + oe‐ACAA2 group than in the sh‐SIRT5 + vector group. Moreover, ACAA2 overexpression partially reversed the upregulation of ACSL4 induced by SIRT5 knockdown (Figure S5H). Consistently, C11‐BODIPY 581/591 staining demonstrated that SIRT5 knockdown exacerbated CaOx‐induced lipid peroxidation, whereas ACAA2 overexpression attenuated the accumulation of lipid peroxides (Figure S5I).

### 3.9 ACAA2 K13 Succinylation Mediates Lipid Remodeling in the CaOx Crystal–Induced Injury Model

**FIGURE 8:**
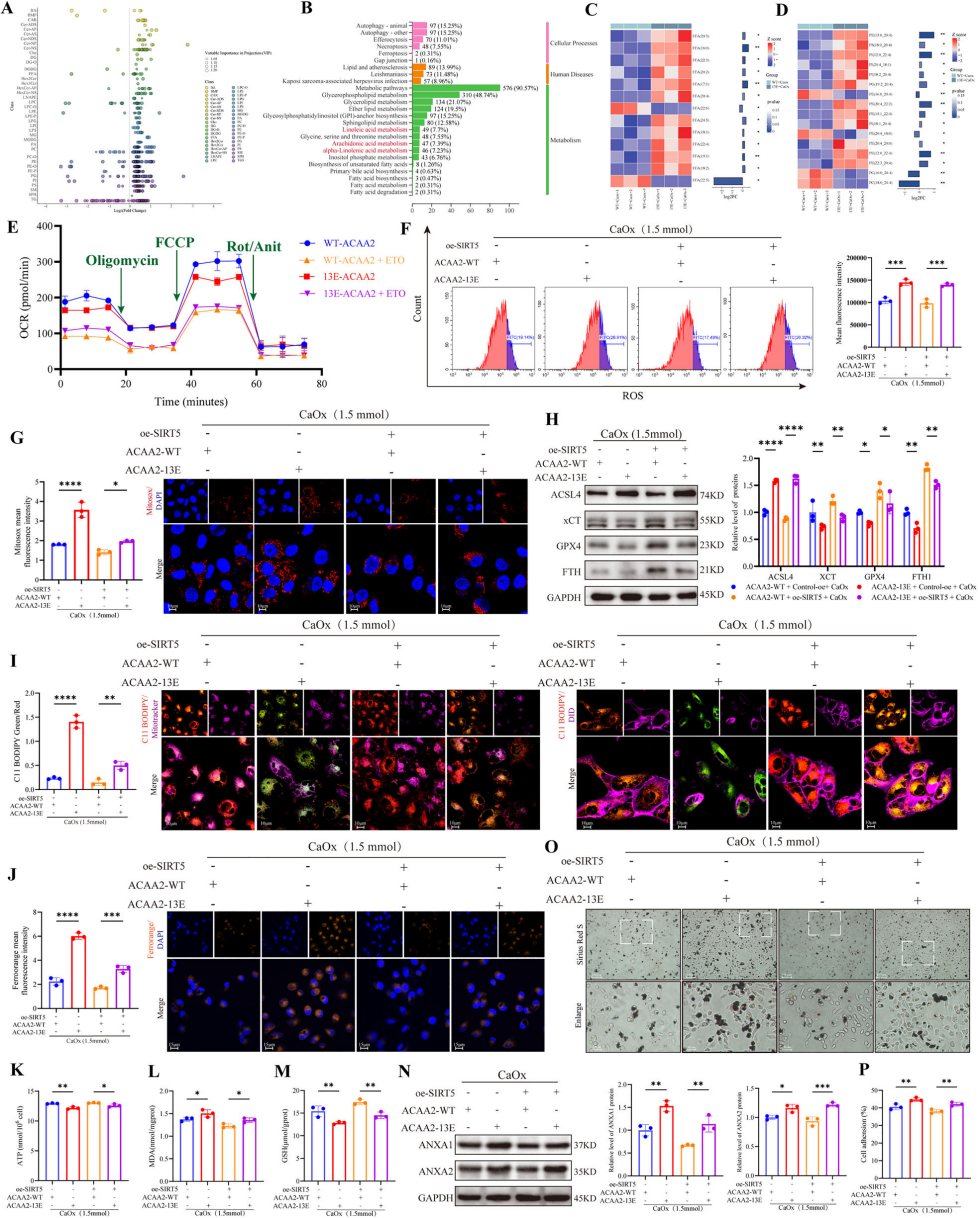
ACAA2 K13 desuccinylation mediates SIRT5 regulation of mitochondrial oxidative injury and ferroptosis in the CaOx crystal‐induced injury model. (A) The forest plot shows the changes in lipid profiles following the ACAA2 K13E point mutation. (B) KEGG enrichment analysis of the differential lipids after the K13E point mutation. (C) The heatmap displays the differential fatty acids following the ACAA2 K13E point mutation. (D) The heatmap shows the differential C20:4‐glycerophospholipids following the ACAA2 K13E point mutation. (E) Seahorse measured OCR profile of ACAA2‐WT and ACAA2 K13E cells with or without etomoxir treatment, followed by injections of mitochondrial respiration inhibitors oligomycin, FCCP, Rot/Anit indicated by arrows. (F) Flowcytometry analysis of ROS levels in the four groups of samples, with quantitative analysis. (G) MitoSOX staining shows the mitochondrial ROS levels in the four groups of samples. (H) Western blot analysis of ACSL4, xCT, GPX4, and FTH expression in the four groups of samples, with quantitative analysis. (I) Co‐staining with C11 BODIPY and Mitotracker or DID reveals lipid peroxidation in the four groups of samples, with quantitative analysis. (J) Intracellular Fe^2^^+^ levels were assessed using the FerroOrange probe in the four groups of samples, with quantitative analysis. (K) ATP levels in the four groups of samples. (L) MDA levels in the four groups of samples. (M) GSH levels in the four groups of samples. (N) Western blot analysis of cell adhesion factors ANXA1 and ANXA2 in the four groups of cells, with quantitative analysis. (O) Representative images show the adhesion of CaOx crystals to HK‐2 cells in the four groups, with corresponding quantitative analysis. (P) Measurement of cell adhesion ability in the four groups. The data represent one set of representative images from independent experiments performed at least three times. Results are expressed as mean SEM. ^*^ *p* < 0.05, ^**^ *p* < 0.01, ^***^ *p* < 0.001, and ^****^ *p* < 0.0001, ns: not significant.

To investigate the impact of succinylation at lysine 13 (K13) of ACAA2 on cellular lipid metabolism, we generated a K13E mutant (lysine‐to‐glutamate substitution) to mimic constitutive succinylation. HK‐2 cells transfected with ACAA2 WT or ACAA2 K13E were subjected to CaOx treatment and analyzed using board target lipidomics profiling. The results showed that the K13E mutation markedly altered the cellular lipid composition, with a total of 574 lipids upregulated and 82 lipids downregulated (Figure S5J,K). Specifically, multiple lipid classes—including bile acids (BAs), FAs, lysophosphatidylcholine (LPC), lysophosphatidylethanolamine (LPE), PC, ether‐linked phosphatidylcholine (PC‐O), PE, ether‐linked phosphatidylethanolamine (PE‐O), PG, and PI—were significantly increased in ACAA2 K13E–transfected cells (Figure 8A). KEGG pathway annotation and enrichment analysis of differential lipids revealed their involvement in multiple cellular processes, including autophagy, gap junctions, ferroptosis, and necroptosis. Within metabolic pathways, glycerophospholipid metabolism was the most prominently enriched, while fatty acid–related pathways such as arachidonic acid metabolism, α‐linolenic acid metabolism, and biosynthesis of unsaturated fatty acids also showed significant enrichment (Figure 8B). Among the differentially expressed FAs, multiple PUFAs, including FA 20:4 and FA 22:4, were significantly elevated in the ACAA2 K13E group (Figure 8C). Furthermore, among the differential glycerophospholipids, various polyunsaturated phospholipids containing 20:4 or 22:4 exhibited a marked upward trend in the ACAA2 K13E group compared with the ACAA2 WT group (Figure 8D). Seahorse extracellular flux analysis was performed to measure the OCR in HK‐2 cells expressing ACAA2 WT or the ACAA2 K13E mutant, and ETO was used to assess cellular FAO capability. The overall OCR was lower in ACAA2 K13E‐expressing cells than in cells expressing ACAA2 WT (Figure 8E). Consistently, ETO‐inhibitable OCR was also reduced in the ACAA2 K13E group compared with the ACAA2 WT group (Figure S5L), indicating that the K13E mutation impairs cellular FAO activity.

### 3.10 ACAA2 K13 Desuccinylation Mediates SIRT5 Regulation of Mitochondrial Oxidative Injury and Ferroptosis in the CaOx ‐Crystalinduced Injury Model

To further investigate the role of ACAA2 K13 desuccinylation in SIRT5‐mediated regulation, HK‐2 cells under CaOx crystal–induced injury were transfected with ACAA2 WT or ACAA2 K13E plasmids in control‐oe and oe‐SIRT5 backgrounds, followed by assessment of mitochondrial oxidative injury and ferroptosis‐related parameters. Total cellular ROS analysis showed that ttransfection of ACAA2 K13E markedly increased ROS accumulation. Compared with the oe‐SIRT5 + ACAA2 WT group, introduction of ACAA2 K13E on the basis of SIRT5 overexpression elevated total ROS levels (Figure 8F). Consistently, in both control‐oe and oe‐SIRT5 cells, ACAA2 K13E expression significantly increased mitochondrial ROS levels compared with ACAA2 WT (Figure 8G). These findings suggest that desuccinylation at ACAA2 K13 is involved in SIRT5‐mediated regulation of mitochondrial oxidative injury. We next evaluated ferroptosis‐related markers. In control‐oe cells, expression of ACAA2 K13E reduced the levels of anti‐ferroptotic proteins GPX4, xCT, and FTH to varying degrees compared with ACAA2 WT. Relative to the control‐oe + ACAA2 WT group, SIRT5 overexpression restored the expression of these anti‐ferroptotic proteins, whereas the ACAA2 K13E mutation partially attenuated this effect. Moreover, ACSL4 expression was decreased in the oe‐SIRT5 + ACAA2 WT group compared with control‐oe + ACAA2 WT, while ACAA2 K13E further reduced ACSL4 levels in the oe‐SIRT5 background (Figure 8H). Assessment of lipid peroxidation revealed that, in control‐oe HK‐2 cells, ACAA2 K13E expression led to greater accumulation of lipid peroxides in both mitochondrial and plasma membrane regions compared with ACAA2 WT. Although SIRT5 overexpression reduced overall lipid peroxidation, cells expressing ACAA2 K13E still exhibited higher lipid peroxidation levels than those expressing ACAA2 WT (Figure 8I). FerroOrange staining further demonstrated that transfection with ACAA2 K13E increased cellular labile Fe^2^^+^ accumulation in both control‐oe and oe‐SIRT5 cells (Figure 8J). In addition, ACAA2 K13E reduced ATP production in both control‐oe and oe‐SIRT5 cells (Figure 8K). In both control‐oe and oe‐SIRT5 cells, ACAA2 K13E mutation promoted greater accumulation of MDA under CaOx stimulation compared with ACAA2 WT (Figure 8L). Furthermore, GSH levels were further decreased in the control‐oe + ACAA2 K13E group compared with control‐oe + ACAA2 WT, and a similar reduction was observed in oe‐SIRT5 cells expressing ACAA2 K13E relative to ACAA2 WT (Figure 8M). Collectively, these data indicate that desuccinylation at ACAA2 K13 contributes to the protective role of SIRT5 against CaOx crystal–induced ferroptosis in RTECs. Plasma membrane lipid peroxidation may enhance cell–crystal adhesion, representing an initiating event in kidney stone formation. Analysis of key adhesion molecules revealed that ACAA2 K13E mutation upregulated the expression of ANXA1 and ANXA2 in both control‐oe and oe‐SIRT5 cells (Figure 8N). Accordingly, we further examined CaOx crystal adhesion in each group. The results showed that, compared with ACAA2 WT, the ACAA2 K13E mutation significantly increased crystal adhesion in both control‐oe and oe‐SIRT5 cells (Figures 8O and S5M). Moreover, Similarly, assessment of cell adhesion capacity revealed that the ACAA2 K13E mutation enhanced the adhesion ability of HK‐2 cells in both control‐oe and oe‐SIRT5 backgrounds (Figure 8P).

### 3.11 Pharmacological Activation of SIRT5 Alleviates CaOx‐Induced Renal Injury and Ferroptosis

Given the critical role of SIRT5 in CaOx crystal‐induced renal injury, we next investigated the therapeutic potential of SIRT5. Mice with Gly‐induced CaOx nephrolithiasis were treated with MC3138 (Figure S6A), a 1,4‐dihydropyridine‐based small‐molecule activator of SIRT5 that has been reported to selectively enhance SIRT5 activity and protect against sepsis‐associated acute kidney injury in mice [19, 20]. Molecular docking analysis predicted a favorable interaction between MC3138 and SIRT5 (Figure S6B). Renal calcium deposition was subsequently evaluated by Von Kossa staining. Compared with the Gly group, MC3138 administration markedly reduced renal calcium deposition (Figure S6C,D). Consistently, TUNEL staining revealed that MC3138 treatment partially reduced renal cell death in mice with CaOx kidney stone (Figure S6E,F). Moreover, renal α‐SMA expression was significantly decreased following MC3138 treatment compared with that in the Gly‐treated group, suggesting an attenuation of renal fibrotic responses (Figure S6G,H). We further examined whether pharmacological activation of SIRT5 affected ferroptosis in CaOx‐induced renal injury. MC3138 treatment partially restored the glyoxylate‐induced reduction in the anti‐ferroptotic protein GPX4 (Figure S6I,J). Consistently, MC3138 markedly attenuated the accumulation of 4‐HNE (Figure S6K,L). Assessment of renal function showed that MC3138 treatment partially attenuated the CaOx‐induced increases in CREA and BUN levels (Figure S6M,N). These findings suggest that SIRT5 represent promising therapeutic targets for CaOx kidney stone.

## 4 Discussion

**FIGURE 9:**
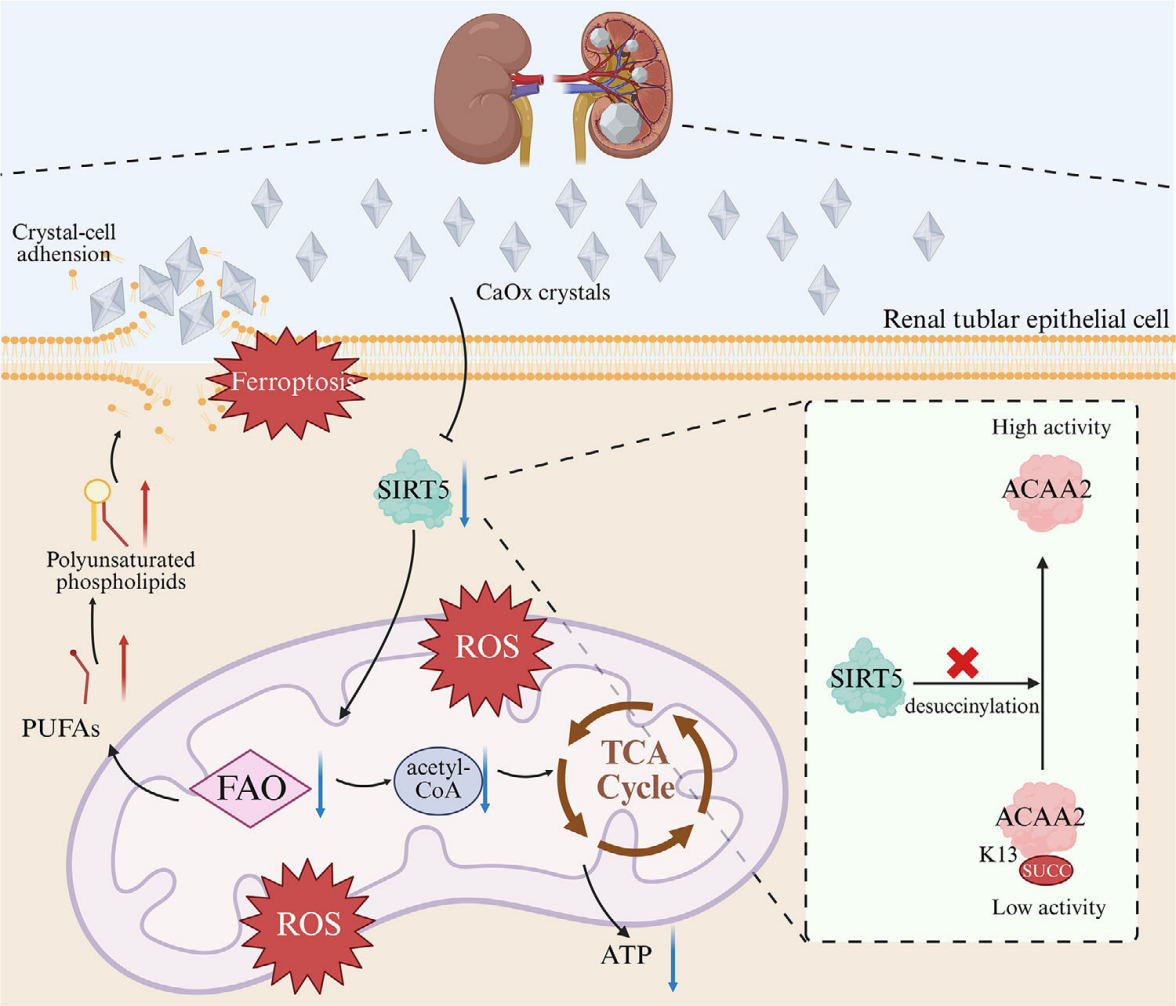
The schematic diagram illustrates the mechanism by which SIRT5 regulates FAO, inhibits mitochondrial oxidative damage, and suppresses ferroptosis in the process of CaOx kidney stone formation in RTEC.

RTECs are metabolically active and highly vulnerable to pathological stress. Under CaOx exposure, metabolic dysregulation and cellular injury in RTECs are closely linked to kidney stone pathogenesis [21]; however, the molecular events governing this process remain incompletely understood. In the present study, we identified a previously unrecognized mechanism by which SIRT5 modulates the activity of ACAA2, a key rate‐limiting enzyme in FAO, through post‐translational modification, thereby mediating CaOx crystal‐induced lipid metabolic dysregulation in renal tissues. Our data demonstrate that CaOx exposure provokes lipid metabolic disturbances in RTECs, which in turn aggravate crystal‐induced cellular injury. Notably, SIRT5 abundance was markedly reduced in RTECs from kidney stone patients and in mouse kidney tissues, and SIRT5 deficiency further exacerbated crystal deposition and renal functional impairment, pointing to a renoprotective role of SIRT5 under CaOx stress. Through integrated multi‐omics profiling, including targeted metabolomics, 4D‐LFQ proteomics, and succinylome analysis, we further showed that SIRT5 deficiency contributes to FAO dysfunction in RTECs, leading to accumulation of PUFAs and subsequent lipotoxicity. These accumulated PUFAs were extensively incorporated into phospholipids, thereby promoting ferroptosis. The resulting oxidative damage to the plasma membrane then provided adhesion sites for CaOx crystals, establishing a self‐perpetuating cycle of crystal‐induced injury, enhanced crystal retention, and progressive cellular destruction. Mechanistically, we identified ACAA2 as a functional substrate of SIRT5‐mediated desuccinylation. Mutation at the K13 residue differentially modulated ACAA2 activity: the K13R substitution inhibited ACAA2 succinylation and enzymatic inactivation, whereas the K13E mutation attenuated the protective effect conferred by SIRT5 overexpression against crystal‐induced injury, thereby exacerbating mitochondrial stress and ferroptosis in RTECs (Figure 9).

Dysregulated lipid metabolism, particularly disturbances in fatty acid metabolism, is increasingly recognized as a central driver of kidney stone initiation and progression. RTECs have exceptionally high energy demands yet limited metabolic flexibility, relying predominantly on FAO for ATP production [21]. Under CaOx exposure, lipid metabolism undergoes complex alterations, among which impaired FAO not only compromises energy supply but also promotes lipid overload and lipotoxicity. Previous studies have reported significantly elevated levels of acetyl CoA and fatty acids in the kidneys of nephrolithiasis model mice, suggesting that FAO dysfunction contributes to stone formation [22]. Recent evidence further indicates that PRMT1 expression is markedly upregulated in RTECs under CaOx stress. Through post‐translational modifications and downstream signaling, PRMT1 promotes PPARγ ubiquitination, thereby suppressing FAO and energy metabolism in RTECs and exacerbating crystal‐induced renal injury [9]. In line with these observations, our study demonstrates that CaOx stress induces substantial PUFA accumulation in RTECs, and excessive unmetabolized PUFAs further aggravate oxidative stress‐mediated cellular damage. Furthermore, FAO and ferroptosis are functionally intertwined, and this interplay has been increasingly implicated in kidney stone pathogenesis. FAO is thought to exert opposing effects on ferroptosis: on one hand, ROS generated during FAO can potentiate lipid peroxidation; on the other hand, FAO‐mediated consumption of PUFAs limits the availability of peroxidizable substrates, thereby suppressing ferroptosis [23, 24]. Nassar et al. reported that DECR1, a key rate‐limiting enzyme of FAO, can influence ferroptosis susceptibility by altering the cellular content of the phospholipids PE and PI [25]. In our study, SIRT5 overexpression enhanced FAO in RTECs, which alleviated PUFA accumulation and polyunsaturated phospholipid buildup induced by CaOx exposure, thereby attenuating ferroptosis. Conversely, SIRT5 knockdown produced the opposite effects. The subcellular localization of lipid peroxidation during ferroptosis is dynamic: it initiates in organellar membranes, including the endoplasmic reticulum, lysosomes, and mitochondria, and subsequently accumulates at the plasma membrane [26]. Notably, modulation of SIRT5 reduced the accumulation of lipid peroxides at the plasma membrane. SIRT5 overexpression reprogrammed the phospholipid composition in RTECs, significantly decreasing PE, PC, and PG species containing 20:4 and 22:4 acyl chains. Given that these phospholipids serve as highly susceptible substrates for peroxidation [27], their reduction diminishes plasma membrane vulnerability to oxidative damage, thereby alleviating CaOx crystal adhesion to RTECs. With the growing recognition of lipid metabolic dysregulation in kidney stone pathogenesis, its therapeutic potential is being actively explored. Lipid metabolism modulators, such as PPAR agonists including fenofibrate and pioglitazone, have been reported to enhance FAO and inhibit stone formation [28, 29]. Statins have also been shown to reduce lipid accumulation and ameliorate tubular injury [9]. Importantly, our study is the first to demonstrate that the SIRT5‐ACAA2 axis mitigates CaOx‐induced renal lipotoxicity. SIRT5 overexpression restores FAO impaired by CaOx crystals, improves cellular energy homeostasis, and alleviates oxidative damage. Meanwhile, selective pharmacological activation of SIRT5 in vivo markedly reduced renal calcium crystal deposition and ameliorated CaOx‐induced renal dysfunction in mice. Collectively, these findings position SIRT5 as a promising therapeutic target for restoring lipid metabolic homeostasis in RTECs and conferring renoprotective effects.

As a post‐translational modification widely present in mitochondrial metabolic enzymes, succinylation exerts complex regulatory effects on diverse cellular metabolic pathways [13]. It is well established that succinylation participates in key energy metabolism processes, including the tricarboxylic acid cycle, ketogenesis, glycolysis, and FAO [15]. SIRT5, one of the most extensively characterized desuccinylases, plays a central role in maintaining cellular energy homeostasis [30]. Accumulating evidence indicates that SIRT5 regulates FAO through post‐translational modifications. Wu et al. demonstrated that in SIRT5 knockout mice, SIRT5 deficiency leads to hyper‐succinylation at the Lys424 site of CPT2, significantly reducing its enzymatic activity, thereby suppressing FAO in cardiomyocytes and exacerbating cardiac lipotoxicity [18]. Other studies have reported that loss of SIRT5 decreases mitochondrial FAO and shifts fatty acid metabolism toward peroxisomes, a redistribution that may exert protective effects under certain contexts [31]. In addition, SIRT5 activates the key FAO enzyme HADHA via desuccinylation at the K644 site, whereas inhibition of SIRT5 exacerbates FAO dysfunction and enhances the cytotoxic effect of venetoclax in acute myeloid leukemia cells [17]. In the present study, multi‐omics analyses, including succinylome profiling, revealed that SIRT5 regulates FAO in RTECs by mediating the desuccinylation of multiple mitochondrial FAO enzymes. These enzymes are involved in several critical steps of FAO, including acyl‐CoA dehydrogenation, enoyl‐CoA hydration, hydroxyacyl‐CoA dehydrogenation, β‐ketothiolysis, and the isomerization and reduction of unsaturated fatty acids. Overexpression of SIRT5 partially restored CaOx‐induced FAO dysfunction, increased intracellular acetyl‐CoA and ATP production in RTECs, and alleviated mitochondrial ROS accumulation and structural damage. SIRT5 is abundantly expressed in RTECs and has been implicated in the development and progression of various kidney diseases [15]. Previous studies have shown that SIRT5 promotes glycolysis and gluconeogenesis while reducing malonylation in non‐mitochondrial metabolic pathways, thereby playing a critical role in metabolic reprogramming in diabetic nephropathy [32]. In renal ischemic injury, SIRT5 deficiency exacerbates mitochondrial structural disruption and functional impairment in RTECs, suggesting a protective role in maintaining mitochondrial integrity [33]. Recent evidence further demonstrates that SIRT5 inhibits ferroptosis in RTECs during sepsis‐induced acute kidney injury by mediating the desuccinylation of the peroxisomal protein PRDX6 [20]. Using renal tubule‐specific SIRT5 knockout mice, our study demonstrates that SIRT5 deficiency exacerbates crystal deposition in the kidney and aggravates crystal‐induced renal injury, ultimately accelerating the loss of renal function in a CaOx kidney stone model.

SIRT5 exerts complex regulatory effects on multiple enzymes involved in different steps of FAO. Among these, we further focused on ACAA2 for in‐depth mechanistic analysis. ACAA2 is a rate‐limiting enzyme that catalyzes the final step of mitochondrial FAO, in which it utilizes free coenzyme A to cleave medium‐ or long‐chain, unbranched 3‐ketoacyl‐CoA, thereby generating acetyl‐CoA [34]. The enzymatic activity of ACAA2 is tightly governed by post‐translational modifications. Previous studies have reported that in triple‐negative breast cancer, ACAA2 activity is modulated by lactylation, and these activity changes influence fatty acid metabolism and subsequent tumor cell proliferation [35]. Han et al. further demonstrated that in non‐alcoholic fatty liver disease, the localization and function of ACAA2 are regulated by palmitoylation; inhibition of this modification activates the AMPK pathway, thereby suppressing hepatic stellate cell activation and suggesting a potential therapeutic value [36]. In the present study, we identified that SIRT5 mediates the desuccinylation of ACAA2 at the K13, K270, and K137 residues, among which succinylation at K13 exerts the most pronounced effect on ACAA2 protein stability and enzymatic activity. Further investigation revealed that the K13E mutation of ACAA2 attenuates the protective effect conferred by SIRT5 overexpression in CaOx‐induced RTEC injury, leading to exacerbated mitochondrial stress and ferroptosis. As a key FAO enzyme, ACAA2 may play a regulatory role in RTEC injury. Previous studies have shown that in acute kidney injury models, the transcription factor KLF15 is significantly downregulated in proximal tubular epithelial cells, and its deficiency suppresses ACAA2 transcription, thereby aggravating RTEC damage [37]. In line with these observations, our findings support a protective role of ACAA2 in the CaOx crystal‐induced injury model, as its overexpression alleviated CaOx‐induced oxidative injury and ferroptosis in RTECs.

Several limitations of the present study should be acknowledged. First, the in vivo experiments were conducted using a Gly‐induced mouse model of CaOx nephrolithiasis. Although this model recapitulates CaOx crystal deposition and the associated renal tubular injury, it does not fully reproduce the chronic, recurrent, and progressive course of human kidney stone disease. Further validation using chronic or recurrent nephrolithiasis models, as well as large‐animal models, are therefore warranted. In addition, the in vitro mechanistic investigations were performed predominantly in HK‐2 cells, and remain to be validated in primary human RTECs. Finally, although the present findings suggest that activation of SIRT5 may alleviate CaOx‐induced renal injury, additional experimental and clinical evidence is required to systematically evaluate the long‐term efficacy, safety, potential off‐target effects, and impact on systemic metabolic homeostasis. Despite these limitations, the present study provides compelling in vitro and in vivo evidence and reveal a previously unrecognized mechanism underlying CaOx crystal‐induced lipid metabolic remodeling in RTECs.

In summary, this study provides the first systematic evidence that the SIRT5‐ACAA2 axis contributes to the maintenance of lipid metabolic homeostasis in RTECs under CaOx stress. Specifically, SIRT5 activates ACAA2 enzymatic activity through desuccinylation, thereby enhancing FAO and alleviating mitochondrial oxidative damage and ferroptosis in RTECs. These findings suggest that SIRT5 may serve as a promising new therapeutic target for preventing or treating CaOx kidney stones.

## Author Contributions

**Qinhong Jiang**: conceptualization, methodology, software, data curation, writing – original draft, investigation, visualization. **Caitao Dong**: methodology, validation, writing – review and editing, visualization, data curation, investigation. **Yuchen Liu**: methodology, investigation, writing – review and editing, data curation. **Yuanquan Lou**: methodology, data curation, software. **Wenlong Lin**: methodology, software, data curation. **Sheng Li**: conceptualization, funding acquisition, writing – review and editing, supervision. **Ziqi He**: supervision, writing – review and editing, funding acquisition, conceptualization, resources.

## Ethics Statement

Our animal experiment protocols were approved by Laboratory Animal Welfare and Ethics Committee of Renmin Hospital of Wuhan University (Approval No. 202400280F).

## Conflicts of Interest

The authors declare no conflicts of interest.

## Supporting information




**Supporting File**: advs77703‐sup‐0001‐SuppMat.docx.

## Data Availability

The data that support the findings of this study are available from the corresponding author upon reasonable request.
