# Supplementary material for: SIRT5‐Mediated Desuccinylation of ACAA2 Protects Against Calcium Oxalate‐Induced Kidney Injury by Regulating Fatty Acid Oxidation to Suppress Mitochondrial Oxidative Injury and Ferroptosis
Source: Adv Sci (Weinh). 2026 Sep 27:e77703. Online ahead of print. doi: 10.1002/advs.77703 (PMC13616336; doi:10.1002/advs.77703)
Supplement: Supplementary file 1 [file ADVS-9999-e77703-s001.docx]

**Table S1: Antibody Information**

| **Antibody** | **Catalog Number** | **Manufacturer** | **Origin** |
| --- | --- | --- | --- |
| SIRT5 | 15122-1-AP | Proteintech | Wuhan, China |
| SIRT5 | 67257-1-Ig | Proteintech | Wuhan, China |
| CD71 | 10084-2-AP | Proteintech | Wuhan, China |
| ACAA2 | 11111-1-AP | Proteintech | Wuhan, China |
| ACAA2 | A15778 | ABclonal | Wuhan, China |
| FTH1 | ab65080 | Abcam | Shanghai, China |
| ACSL4 | A20414 | ABclonal | Wuhan, China |
| ANXA2 | A11235 | ABclonal | Wuhan, China |
| ANXA1 | 21990-1-AP | Proteintech | Wuhan, China |
| DYKDDDDK tag | 66008-4-Ig | Proteintech | Wuhan, China |
| HA Tag | M20003 | Abmart | Wuhan, China |
| xCT | DF12509 | Affinity Biosciences | Hangzhou, China |
| GPX4 | 67763-1-Ig | Proteintech | Wuhan, China |
| succK | PTM-401 | PTM Biolab | Hangzhou, China |
| succK | PTM-419 | PTM Biolab | Hangzhou, China |
| CPT1A | 15184-1-AP | Proteintech | Wuhan, China |
| 4-HNE | 68538-1-Ig | Proteintech | Wuhan, China |
| NQO-1 | 11451-1-AP | Proteintech | Wuhan, China |
| HMOX1 | 10701-1-AP | Proteintech | Wuhan, China |
| NGAL | 31721-1-AP | Proteintech | Wuhan, China |
| KIM-1 | 83221-2-RR | Proteintech | Wuhan, China |
| αSMA | 14395-1-AP | Proteintech | Wuhan, China |
| Ki-67 | 27309-1-AP | Proteintech | Wuhan, China |
| GAPDH | 81640-5-RR | Proteintech | Wuhan, China |

**Supplementary Figure S1.** (A) CCK-8 assay was used to measure the cell viability of HK-2 cells under different CaOx concentration gradients. (B) CCK-8 assay was used to measure the cell viability of HK-2 cells under different CaOx time gradients. (C) Total ion current chromatogram from mass spectrometry analysis of the quality control samples. (D) Forestplot shows changes in lipid profiles of HK-2 cells under CaOx crystal intervention. (E) Bubble plot depicting the most significantly different lipids. The data represent one set of representative images from independent experiments performed at least three times. Results are expressed as mean ± standard error of the mean (SEM). ^*^*P* < 0.05, ^**^*P* < 0.01, ^***^*P* < 0.001, and ^****^*P* < 0.0001; ns, not significant.

**Supplementary Figure S2.** (A, B) Western blot analysis confirms the efficiency of SIRT5 overexpression, with corresponding quantitative analysis. (C, D) Western blot analysis confirms the efficiency of SIRT5 knockdown, with corresponding quantitative analysis. (E) DNA gel electrophoresis for genotyping mice with renal tubular epithelial cell-specific Sirt5 knockout. (F, G) Western blot analysis of SIRT5 expression in the kidneys of renal tubular epithelial cell-specific Sirt5 knockout mice, with quantitative analysis. (H) Representative images show the distribution of peptides identified by mass spectrometry. (I) Heatmap illustrates the Pearson correlation coefficients between all samples. (J) Violin plot displays the within-group and between-group data variability. (K) Total ion current chromatogram from mass spectrometry analysis of the quality control samples. (L) PCA plot shows the intracellular lipid distribution following SIRT5 overexpression. The data represent one set of representative images from independent experiments performed at least three times. Results are expressed as mean ± SEM. ^*^*P* < 0.05, ^**^*P* < 0.01, ^***^*P* < 0.001, and ^****^*P* < 0.0001; ns, not significant.

**Supplementary Figure S3.** (A) SMPDB enrichment analysis of the differential lipids. (B) Intracellular levels of O-acetylcarnitine in the two groups of cells. (C) Volcano plot shows the differential proteins after SIRT5 overexpression. (D) Heatmap displays the protein distribution following SIRT5 overexpression. (E) Quantitative analysis of CPT1A protein expression in cells from each group. (F) Quantitative analysis of CPT1A fluorescence intensity in the kidney tissues from the four groups of mice. (G, H) Flowcytometry analysis of ROS levels in each group of cells, with corresponding quantitative analysis. (I) JC-1 staining was used to assess mitochondrial membrane potential in cells from each group, with corresponding quantitative analysis. The data represent one set of representative images from independent experiments performed at least three times. Results are expressed as mean ± SEM. ^*^*P* < 0.05, ^**^*P* < 0.01, ^***^*P* < 0.001, and ^****^*P* < 0.0001; ns, not significant.

**Supplementary Figure S4.** (A) Western blot analysis shows the expression levels of ACSL4, xCT, GPX4, and FTH in cells from four groups. (B) Quantitative analysis of ACSL4, xCT, GPX4, and FTH protein expression in cells from four groups. (C) The lipid peroxidation was assessed by co-staining C11 BODIPY in cells from four groups. (D) Quantitative analysis of ACSL4, xCT, GPX4, and FTH protein expression in the kidneys of the four groups of mice by Western blot. (E) Quantitative analysis of GPX4 fluorescence intensity in kidney tissues of the four groups of mice. (F) Quantitative analysis of HNE-4 fluorescence intensity in kidney tissues of the four groups of mice. (G) Quantitative analysis of FerrOrange staining in cells from each group. (H) Quantitative analysis of ANXA1 and ANXA2 protein expression in cells following SIRT5 overexpression by Western blot. (I) Quantitative analysis of ANXA1 and ANXA2 protein expression in cells following SIRT5 knockdown by Western blot. (J) The cell adhesion capacity of each group was evaluated. The data represent one set of representative images from independent experiments performed at least three times. Results are expressed as mean ± SEM. ^*^*P* < 0.05, ^**^*P* < 0.01, ^***^*P* < 0.001, and ^****^*P* < 0.0001; ns, not significant.

**Supplementary Figure S5.** (A) Western blot analysis of SIRT5 and ACAA2 protein expression under different CaOx time gradient interventions. (B) Western blot analysis of FLAG expression to verify the transfection of point mutation plasmids. (C) Western blot analysis of NQO1 and HMOX1 protein expression and quantitative analysis in cells from the four groups. (D) CCK-8 assay measuring cell viability in the four groups. (E) Measurement of T-AOC in cells from the four groups. (F) Measurement of SOD levels in cells from the four groups. (G) Measurement of LDH levels in cells from the four groups. (H) Western blot analysis of ACSL4, xCT and GPX4 protein expression, with corresponding quantitative analysis. (I) The lipid peroxidation was assessed by co-staining C11 BODIPY in cells from four groups. (J) Volcano plot showing differential lipids following the ACAA2 K13E point mutation. (K) PCA plot showing the intracellular lipid distribution following the ACAA2 K13E point mutation. (M) Quantitative analysis of C11 BODIPY staining in cells from each group. The data represent one set of representative images from independent experiments performed at least three times. Results are expressed as mean ± SEM. ^*^*P* < 0.05, ^**^*P* < 0.01, ^***^*P* < 0.001, and ^****^*P* < 0.0001; ns, not significant.

**Supplementary Figure S6.** (A) Schematic diagram of the animal experimental workflow. (B) Molecular docking analysis shows the binding interaction between SIRT5 and MC3138. (C) Von Kossa staining illustrates calcium salt deposition in the kidneys of the four groups, with (D) quantitative analysis. (E) Representative images of TUNEL staining in kidney tissues from the four groups are shown, with (F) quantitative analysis. (H) Immunofluorescence staining demonstrates the expression of α-SMA in kidney tissues from the four groups are shown, with (G) quantitative analysis. (I) Immunofluorescence staining demonstrates the expression of GPX4 in kidney tissues from the four groups are shown, with (J) quantitative analysis. (K) Immunofluorescence staining demonstrates the expression of GPX4 in kidney tissues from the four groups are shown, with (L) quantitative analysis. (M) Serum CREA levels were measured in the four groups of mice. (N) Serum BUN levels were measured in the four groups of mice. The data represent one set of representative images from independent experiments performed at least three times. Results are expressed as mean ± SEM. ^*^*P* < 0.05, ^**^*P* < 0.01, ^***^*P* < 0.001, and ^****^*P* < 0.0001; ns, not significant.
